# Direct and indirect effects of 13-valent pneumococcal conjugate vaccine on pneumococcal carriage in children hospitalised with pneumonia from formal and informal settlements in Mongolia: an observational study

**DOI:** 10.1016/j.lanwpc.2021.100231

**Published:** 2021-07-30

**Authors:** Jocelyn Chan, Tuya Mungun, Purevsuren Batsaixan, Mukhchuluun Ulziibayar, Bujinlkham Suuri, Dashpagam Otgonbayar, Dashtseren Luvsantseren, Cattram D Nguyen, Dorj Narangarel, Eileen M Dunne, Kimberley Fox, Jason Hinds, Monica L Nation, Casey L Pell, E Kim Mulholland, Catherine Satzke, Claire von Mollendorf, Fiona M Russell, PneuCAPTIVE Mongolia Research Group

**Affiliations:** 1 Infection and Immunity, Murdoch Children's Research Institute (MCRI), Melbourne, Australia; 2 Department of Paediatrics, University of Melbourne, Melbourne, Australia.; 3 National Centre for Communicable Diseases (NCCD), Ministry of Health, Ulaanbaatar, Mongolia.; 4 Regional Office for the Western Pacific, World Health Organization, Manila, Philippines.; 5 Institute for Infection and Immunity, St George's University of London, London, United Kingdom.; 6 BUGS Bioscience, London Bioscience Innovation Centre, London, United Kingdom.; 7 Department of Infectious Disease Epidemiology, London School of Hygiene and Tropical Medicine, London, United Kingdom.; 8 Department of Microbiology and Immunology, University of Melbourne at the Peter Doherty Institute for Infection and Immunity, Melbourne, Australia.

**Keywords:** Pneumococcal conjugate vaccines, herd immunity, indirect effects, pneumococcal carriage, informal settlements, Mongolia, vaccine coverage

## Abstract

**Background:**

Within Ulaanbaatar, Mongolia, risk factors for pneumonia are concentrated among children living in informal settlements comprised of temporary shelters (gers). We used pneumococcal carriage surveillance among children from formal and informal settlements hospitalised with pneumonia to evaluate the direct and indirect effects of 13-valent pneumococcal conjugate vaccine (PCV13) against vaccine-type (VT) pneumococcal carriage following a phased introduction of PCV13.

**Methods:**

We enrolled and collected nasopharyngeal swabs from children 2-59 months of age presenting to hospital. Pneumococci were detected using lytA qPCR and serotyped using microarray on a random monthly selection of swabs between November 2015 and March 2019 from two districts in Ulaanbaatar. PCV13 status was determined using written records. We quantified the associations between individual PCV13 status (direct effects) and district-level PCV13 coverage (indirect effects) and VT carriage using generalised estimating equations and explored interactions by settlement type.

**Findings:**

A total of 1 292 swabs from 6 046 participants were tested for pneumococci. Receipt of PCV13 and increasing PCV13 coverage independently reduced the risk of VT carriage. For each percent increase in PCV13 coverage, the adjusted odds of VT carriage decreased by 1•0% (OR 95% CI 0•983-0•996; p=0•001), with a predicted decrease in VT carriage rate from 29•1% to 13•1% as coverage reached 100%. There was a trend towards a slower decline within informal settlements (p=0•100). Adjusted PCV13 vaccine effectiveness against VT carriage was 39•1% (95% CI 11•4-58•1%, p=0•009).

**Interpretation:**

Substantial indirect effects were observed following PCV13 introduction, including among children living within informal settlements.

**Funding:**

Bill & Melinda Gates Foundation; Gavi, the Vaccine Alliance.

**Research in context**

Evidence before this study

We searched PubMed for studies evaluating the relationship between pneumococcal conjugate vaccine (PCV) coverage and indirect effects published between January 2001 and January 2021. We used the terms “pneumococcal conjugate vaccine”, “indirect”, “herd” and “vaccine coverage”. We further supplemented the search with expert input from co-authors to identify any relevant studies. We identified a total of five studies examining the association between PCV coverage and indirect effects on either disease (n=3) or vaccine-type (VT) carriage (n=2). All studies describe an association between PCV coverage and pneumococcal disease or VT carriage. Only one of these research studies was conducted in either a low- or middle-income country – Brazil. This study found that higher uptake of PCV10 within a mesoregion was associated with fewer pneumonia hospitalisations among children aged less than 12 months (marginal mean rate ratio [RR] 0•83; 95% CI 0•72-0•96) and among adults aged 18–39 years (marginal mean RR 0•81; 95% CI 0•70-0•93). One of the studies was a meta-analysis of 70 studies, including seven middle-income countries. This meta-analysis found greater reductions in disease due to PCV7 serotypes in settings with higher PCV coverage (per percentage; RR 0•995, 95% credible interval 0•989-0•998). None of the studies adjusted for individual-level confounders.

The two studies from the United States indicated that moderate levels of PCV coverage were sufficient for substantial indirect effects. However, both studies were ecological in study design. In contrast, carriage surveys from Malawi, which did not specifically examine the relationship between PCV coverage and VT carriage, showed high levels of circulating VT carriage despite high levels of vaccine coverage seven years post-PCV introduction, indicating that further research using more robust study designs is required in a range of settings.

Added value of this study

Our research addresses a critical gap in our understanding of the relationship between PCV coverage and indirect effects. Our findings show that in Mongolia, which introduced 13-valent PCV (PCV13) and included a catch-up campaign for children up to two years of age, indirect effects were observed at low levels of PCV13 coverage and increased as vaccine coverage increased.

Our results also help to explain why control of VTs have been challenging in settings with high rate of VT carriage, despite high rates of vaccine coverage. While we observed indirect effects in children across both formal and informal settlements in Mongolia, the association between PCV coverage and indirect effects differed by settlement type. There was a trend towards greater indirect effects at lower levels of coverage for children living in apartment subdistricts (formal), compared with children living in ger subdistricts (informal) and mixed subdistricts, who have a higher rate of VT carriage. Hence, our results support dynamic modelling studies demonstrating that higher rates of vaccine coverage are required to effectively interrupt person-to-person transmission in populations with high prevalence and transmission of VTs.

Implications of all the available evidence

Our results demonstrate indirect effects following PCV13 introduction in Mongolia, supporting the inclusion of indirect effects in a previous economic model in Mongolia which found PCV to be highly cost-effective, reducing disease costs by US$440 000 in the first year of PCV introduction. These results have relevance for other countries in the region, many of which have not yet introduced PCV.

Globally, there is strong interest in reducing the number of PCV doses (from three or four doses to two doses) to minimise program costs once VTs have been controlled. However, reduced-dose schedules rely on sufficient indirect effects to maintain vaccine impacts. Not all countries have surveillance systems capable of accurately measuring indirect effects on disease. This study demonstrates how pneumococcal carriage surveillance can be used to monitor the control of VTs and potentially be applied to monitor the maintenance of indirect effects following any schedule change. Our findings also contribute ne insight into conditions necessary to generate indirect effects – demonstrating that higher PCV coverage may be required in settings with high prevalence of VTs. Indeed, high vaccination coverage alone may not be sufficient to control VTs in settings with very high prevalence of VT pneumococci. Alternative schedules, or wider catch-up programs encompassing children over five years of age may be required to achieve control of VTs. Further research is needed to understand social and environmental conditions contributing to high prevalence of VT pneumococci and to develop suitable vaccination strategies for these settings.

Alt-text: Unlabelled box

## 1 Introduction

Pneumococcal disease, including meningitis, septicaemia and pneumonia, is a leading cause of child morbidity and mortality globally, and the burden is not evenly distributed across or within countries.^1^ Pneumococcal disease is preceded by nasopharyngeal pneumococcal carriage, and transmission of pneumococci occurs through close contact with carriers.^2^ Risk factors such as malnutrition, air pollution and overcrowding concentrate the disease among the most marginalised children within societies.^3^^,^^4^ Pneumococcal conjugate vaccines (PCVs) have substantially reduced a large global burden of disease due to Streptococcus pneumoniae in many settings.^1^ The vaccine provides both direct effects to vaccinated children and indirect effects to the wider population.^5^ Reducing the number of people who are susceptible to vaccine-type (VT) pneumococcal carriage through vaccination results in reduced of transmission VT pneumococci – protecting both vaccinated and unvaccinated individuals in that population.^6^ These are known as the indirect or herd effects of a vaccine.

Indirect effects extend the benefits of the vaccine to unvaccinated groups, including infants too young to be vaccinated and the elderly.^5^ Furthermore, indirect effects are critical to achieve control of VTs – a prerequisite for the introduction of reduced dose schedules. Reduced dose schedules aim to maintain vaccine impact while reducing PCV program costs through the use of only two doses (as opposed to three or four doses).^7^ In particular, reduced-dose schedules rely on the continued generation of indirect effects to protect infants who have not received sufficient doses to generate individual protection, but are highly susceptible to disease.^7^

Respiratory diseases, including pneumonia, are the second largest cause of under-five mortality in Mongolia.^8^ Prior to PCV introduction, there was a high incidence of all-cause pneumonia hospitalisations among children 12-59 months of age, at an estimated 31•8 per 1 000 population^9^ High levels of air pollution and extremes of cold temperature in winters contribute to a seasonally high burden of pneumonia.^10^ While local data on the aetiology of childhood pneumonia is limited, modelled estimates of pneumococcal pneumonia incidence (19•8 per 1 000 population [95% credible interval 17•1-23•6]) suggest that the pneumococcus is responsible for a large proportion of the overall pneumonia burden in Mongolia. ^1^

Forty-five percent of the Mongolian population resides in the capital city of Ulaanbaatar.^11^ Rapid urbanisation has led to the growth of informal settlements comprising of ger dwellings (traditional temporary shelters) clustered in particular subdistricts (ger subdistricts). Within ger subdistricts, there are issues of household crowding and air pollution (from burning coal for heating), which are risk factors for pneumonia and pneumococcal disease.^3^^,^[12], [13], [14] From the latest census, 30.4% of the city's population live in gers. These health and socioeconomic inequalities are characteristic of many urban settings in the Asia-Pacific region.^15^ There is no evidence on the impact of PCVs within informal settlements.

Mongolia was among the first countries in Asia to introduce PCV. ^16^ The phased introduction of 13-valent PCV (PCV13) commenced in 2016 and is being evaluated through enhanced pneumonia surveillance.^17^ Monitoring the indirect effects of PCVs can be challenging.^18^ The recommended method for evaluating PCV impact – invasive pneumococcal disease (IPD) surveillance – requires large number of samples to detect a rare outcome (IPD).^19^ Alternatively, surveillance of pneumococcal carriage can be easier to establish and is well-suited to monitoring indirect effects, since indirect effects are mediated by reductions in VT carriage.^18^ Community carriage surveys conducted before and one year after PCV13 introduction found a 51% reduction in VT carriage (adjusted prevalence ratio 0.49 [95% CI 0.33–0.73]) in unvaccinated infants aged 5-8 weeks, indicating the presence of indirect effects.^20^

Indirect effects are traditionally measured by comparing the incidence of disease among unvaccinated individuals before and after vaccine introduction, since unvaccinated individuals are reliant on indirect effects for protection. However, in this study we adapted a method pioneered by Ali et al, which evaluates indirect effects by assessing correlations between levels of vaccine coverage and vaccine-related outcomes (VT carriage) among both vaccinated and under-vaccinated individuals, since both groups benefit from indirect effects.^21^ Conducting ongoing carriage surveillance among children hospitalised with pneumonia allowed us to characterise gradual changes in carriage patterns and explore the relationship between PCV13 coverage and vaccine impact. Furthermore, carriage surveillance among this cohort is more likely to represent the serotypes responsible for disease, compared to studies conducted among healthy children in the community.

In Mongolia, we hypothesise that VT carriage will decline post-PCV13 introduction and that the risk of VT carriage declines both as district-level PCV13 coverage rises and by individual PCV13 vaccination status. Using carriage surveillance among children hospitalised with pneumonia in Ulaanbaatar, we aim to: 1) describe trends in VT carriage pre- and post-PCV13 introduction, 2) describe the relationship between district-level PCV13 coverage and risk of VT carriage (i.e., indirect effects), and 3) calculate the PCV13 vaccine effectiveness (VE) (i.e., direct effects) against VT carriage. As part of Aim 2, we also explore an interaction between PCV13 coverage and subdistrict type (apartment, ger or mixed housing).

## 2 Methods

### 2.1 Study setting

Ulaanbaatar has a population of approximately 1•3 million people, divided into nine districts and 121 subdistricts.^11^ This study was conducted among children resident in two districts (Songinokhairkhan and Sukhbaatar) in Ulaanbaatar – the first to receive PCV13 as part of a phased introduction in June 2016. PCV13 was introduced in one additional district (Bayanzurkh) one year later and rolled out across the rest of Ulaanbaatar in March 2018. Children received a “2+1” schedule given at two, four and nine months of age. A three-month catch-up campaign, comprised of two doses for children 3-23 months of age, accompanied vaccine introduction in the first three districts.^17^ Immunisations are available at no cost through family health centres to which children are registered based on their subdistrict of residence. PCV coverage among the target age group within the PCV intervention districts was reported to be 97-98% from 2017-2019.^22^ Mongolia does not have an adult pneumococcal vaccination program.

### 2.2 Study design, participant recruitment and data collection

This observational study was embedded within an enhanced hospital-based pneumonia surveillance system which commenced in April 2015, modified from the World Health Organization Invasive Bacterial Vaccine Preventable Disease (WHO IB-VPD) Surveillance, and conducted in partnership between the Murdoch Children's Research Institute (MCRI), the WHO and the Mongolian Ministry of Health.

Participants with pneumonia were recruited from four participating district hospitals and the tertiary Maternal and Child Health Hospital according to a previously published protocol.^17^ These hospitals represent the entirety of public hospital care for the two study districts. Most pneumonia cases are likely to be captured by the public hospital system, since the vast majority of healthcare for children is provided by the public sector, with few children accessing private care. The two study districts, Songinokhairkhan and Sukhbaatar, comprise approximately 36% of the city's population and are broadly representative of Ulaanbaatar.^11^^,^^17^ In brief, hospital staff members identify admissions that meet the eligibility criteria, complete a standard case report form (CRF) and collect relevant specimens. Participants were eligible if they resided within the two study districts included in the initial phased PCV13 introduction, were aged 2-59 months, and presented with cough or difficulty breathing, and either a respiratory rate ≥ 50 breaths per minute, hypoxia (oxygen saturation < 90%) or a clinical diagnosis of severe pneumonia.^17^

We collected information on demographics, clinical presentation, dates of PCV administration, antibiotics received and risk factors for pneumococcal carriage (see Supplementary Table 3). PCV13 status was determined using written records from parent-held child health cards and validated against an electronic immunisation register.^23^ PCV13 vaccination status was based on documented PCV13 doses at least 14 days before enrolment. Participants were defined as ‘vaccinated’ if they received an adequate number of doses for immune protection (i.e., two or more PCV13 doses at <12 months of age or at least one dose after the age of 12 months). Under-vaccinated participants included those who had received no doses.^18^ We classified each child's residence into either formal (houses and apartments) or informal (gers) housing. Classification of subdistricts based on the predominant housing type were provided by the City Health Department; the categories were 1) apartments, 2) gers or 3) mixed subdistricts (combination of apartments, houses and gers).

From each participant, we collected a single nasopharyngeal sample on admission using a paediatric flocked swab. Where possible, the sample was obtained prior to antibiotic administration, however antibiotic status at the time of swab collection was collected as part of the CRF. We stored the sample in 1ml of skim milk tryptone glucose glycerol (STGG) medium then transported it to the National Centre for Communicable Disease laboratories (Ulaanbaatar, Mongolia).^24^ We tested for pneumococcal carriage among participants enrolled six months pre-PCV13 introduction and up to two years and 10 months post-PCV13 introduction.^5^ To achieve our sample size of 1 200 (see sample size section below), a random sample of 33 swabs were selected for testing each month. Random samples were selected by sorting on random numbers generated using the runiform function in Stata 15. Similar methods were employed as part of a multi-site study to examine the relationship between PCV13 coverage and indirect effects.^18^

Participant data were double-entered using electronic databases. Regular double-entry discrepancy and logic checks were conducted prior to analysis.

### 2.3 District vaccination coverage

Data on monthly number of PCV13 doses administered within each district were collated by staff from the Mongolian Expanded Programme of Immunizations team (Supplementary Tables 1 and 2). These data were collected from written registration books at family health centres (Supplementary Methods page 1) which documented personal identifiers and dates of PCV13 administration. Population data estimates were provided by the Mongolian Department of Health for 2018. Monthly district-level vaccination coverage was calculated as the number of children who had received at least two doses of PCV13 divided by the total number of children under five years within each district. Two doses are considered sufficient to offer protection for infants and at least one dose in children over 12 months of age.^25^^,^^26^ However, date of birth was not available to calculate exact age from the routine administrative immunisation registers so we defined any child under five who had received two doses of PCV13 to be vaccinated.

### 2.4 Laboratory methods

Nasopharyngeal samples were vortexed and dispensed into aliquots for freezing at ultra-low temperatures (≤-70°C). Samples were shipped on dry ice to the Murdoch Children's Research Institute (Melbourne, Australia) for sample testing and analysis according to standard methods.^24^ Testing was completed following an initial freeze-thaw. Laboratory staff screened for the presence of pneumococci using quantitative real-time PCR (qPCR) targeting the lytA gene.^27^ Molecular serotyping was performed using Senti-SP v1.5 microarray (BUGS Bioscience) as previously described.^28^^,^^29^ Samples that were lytA qPCR positive (Ct value < 35) but not able to be serotyped (either culture negative or due to technical difficulties) were considered pneumococcal positive, serotype unknown. For more details see previously published methods.^27^

We defined VT carriage as carriage of any pneumococcal serotype included in the PCV13 vaccine; i.e. serotypes 1, 3, 4, 5, 6A, 6B, 7F, 9V, 14, 18C, 19A, 19F and 23F. Samples were considered VT carriage positive if they contained at least one PCV13 serotype, regardless of any other serotypes present in the sample. Overall carriage was defined as carriage of any pneumococcal serotype.

### 2.5 Analyses

Participant characteristics were presented by vaccination status, subdistrict type and year to identify trends over time as vaccine coverage increased. Trends in VT carriage over time were graphed among vaccinated and under-vaccinated participants (Aim 1). We calculated moving carriage rates within 5-month rolling intervals to smooth random variation due to small monthly participant numbers. Rates were adjusted by age group (2-11 months, 12-23 months, 24-59 months) since age is a known determinant of pneumococcal carriage. We used direct adjustment to account for random variation in the age of participants recruited each month.^30^ PCV13 vaccination coverage in the two study districts was also graphed over time.

Our primary analyses (Aim 2) examined the relationship between district-level PCV13 coverage among children under five years of age and risk of VT carriage. We first ascertained for each participant the district-level PCV13 coverage at their district of residence within the month of their hospital admission.

Since participants from the same subdistrict are not independent, we used generalised estimating equations (with a binomial distribution, a logit link function, and an exchangeable correlation structure) to account for clustering at the subdistrict level. We included participants with complete data for all variables in the model (complete case analysis). We adjusted by PCV13 vaccination status, season (month of swab collection), age group, subdistrict type (ger, mixed, apartment), housing type (formal or informal), maternal education, household income, household crowding (greater than three people per room), number of children under five years of age, cigarette exposure, household fuel type, and antibiotic exposure (Supplementary Table 3). These covariates were selected a priori using a directed acyclic graph (DAG), informed by relevant literature and refined through expert consultation (Supplementary Figure 1). The linearity assumption was tested using a lowess plot. To present the results of the above model, we estimated and graphed the marginal mean VT carriage rate by decile of PCV13 coverage, accounting for the balance of the other covariates across all individuals. Marginal mean VT carriage rate was determined using the margins and marginsplot commands in Stata. Since PCV13 status was included as a covariate in the adjusted model, we were able to calculate adjusted vaccine effectiveness (VE) as one minus the odds ratio of PCV13 carriage in vaccinated versus under-vaccinated children multiplied by 100 (Aim 3).

We conducted an additional analysis exploring an interaction between PCV13 coverage and subdistrict-type since we hypothesized that children living in ger subdistricts would be at higher risk of pneumococcal carriage and higher coverage may be required to achieve indirect effects in this setting. We chose to include subdistrict-type as an interaction term rather than individual-level housing type since indirect effects are generated at a community rather than individual level.

To act as a bias indicator, we developed an additional model using overall pneumococcal carriage as the outcome, since most studies indicate that PCV13 does not affect overall pneumococcal carriage (due to replacement with non-VTs).^18^^,^^31^ Since hospital-based carriage surveillance among children with pneumonia may be affected by prior antibiotic use, we also presented supplementary data on the presence of antimicrobial resistance (AMR) genes to assess the validity of our findings.

All analyses were undertaken using Stata 15.^32^ The community-contributed command baselinetable was used to construct Table 1.^33^

### 2.6 Sample size

Sample size calculations were based on Aim 2. As described in the published protocol, calculations performed using nQuery Advisor+nTerim 4.0 were based on sample size methods for logistic regression models with a continuous covariate (i.e., PCV13 coverage) with inflation to account for clustering within subdistricts (intraclass coefficient of 0.1).^27^ We assumed a VT carriage rate of 30% at the mean PCV13 coverage level, and a VT carriage rate of 20% at one standard deviation above the mean. Assuming a significance level of 0.05, allowing for adjustment using multiple covariates with an R^2^ of 0.4, 600 under-vaccinated participants would provide 87% power to detect the association between PCV13 coverage and VT carriage.

### 2.7 Ethics approvals

This study was conducted according to the protocols approved by the Mongolian National Ethics Committee for Health Research, The Royal Children's Hospital (33177B) and the WHO Regional Office for the Western Pacific (WPRO) Ethics Research Committee (2013.30.LAO.2.EPI).

### 2.8 Role of the funding source

This work is supported by the Bill & Melinda Gates Foundation (grant number OPP1115490) and the Gavi Alliance (contract number PP61690717A2). JC is funded by the Australian Government Research Training Program scholarship. FMR is funded by a NHMRC Investigator grant. CS was supported by an Australian NHMRC Career Development Fellowship (1087957) and a Veski Inspiring Women Fellowship. MCRI was supported by the Victorian Government's Operational Infrastructure Support Program. The funders had no role in study design, data collection and analysis, decision to publish, or preparation of the manuscript.

## 3 Results

### 3.1 Participant characteristics

Between November 2015 and March 2019, we enrolled 6 049 children 2-59 months of age (inclusive) and tested a random sample of 1 283 participants for pneumococcal carriage (median 32•5 sampled per month, interquartile range 32•0-33•0 per month). The 1 283 participants were representative of the overall participants enrolled, with the exception of season, since enrolment of participants with pneumonia greatly increased during the cold season, while monthly random sampling did not change over time (Supplementary Table 4).

Among the participants who were under-vaccinated, 81•2% received no doses. Compared with vaccinated children, under-vaccinated children were more likely to carry PCV13 serotypes (Table 1). The characteristics of participants that were tested for pneumococcal carriage were consistent over time, although participants enrolled from the two districts varied over time (Supplementary Table 5 and Supplementary Figure 3). Pneumococcal risk factors, including household crowding and use of wood or coal for fuel, and pneumococcal carriage were higher among children from ger and mixed subdistricts than children from apartment subdistricts (Table 2). The proportion of missing data was less than 10% for all variables including PCV13 status (7•5% missing).

**Table 1:** Characteristics of participants tested for pneumococcal carriage by vaccination status*, Ulaanbaatar, Mongolia, November 2015-March 2019

	Under-vaccinated	Vaccinated	Missing vaccination status	Total
	N=677	N=509	N=97	N=1 283

Age (months) (N=1 283) †	15 (6-28)	15 (9-22)	17 (8-26)	15 (7-25)
Gender (N=1 283)				
Female	319 (47•1%)	217 (42•6%)	33 (34•0%)	569 (44•3%)
Male	358 (52•9%)	292 (57•4%)	64 (66•0%)	714 (55•7%)
Season‡ (N=1 283)				
Warm	332 (49•0%)	265 (52•1%)	77 (79•4%)	674 (52•5%)
Cold	345 (51•0%)	244 (47•9%)	20 (20•6%)	609 (47•5%)
Household income§ (N=1 206)				
At or below minimum	303 (48•3%)	220 (44•9%)	36 (40•4%)	559 (46•4%)
Above minimum	324 (51•7%)	270 (55•1%)	53 (59•6%)	647 (53•6%)
Maternal education (N=1 261)				
Tertiary	337 (50•7%)	258 (51•6%)	46 (47•9%)	641 (50•8%)
Secondary school or less	328 (49•3%)	242 (48•4%)	50 (52•1%)	620 (49•2%)
Crowding (N=1 244)				
<=3 people per room	356 (54•0%)	273 (55•4%)	53 (57•6%)	682 (54•8%)
>3 people per room	303 (46•0%)	220 (44•6%)	39 (42•4%)	562 (45•2%)
Other children aged <5 years in the house (N=1 245)				
None	431 (65•5%)	345 (69•3%)	62 (69•7%)	838 (67•3%)
At least one child	227 (34•5%)	153 (30•7%)	27 (30•3%)	407 (32•7%)
Smoker in the house (N=1 263)	304 (45•6%)	210 (41•9%)	31 (32•3%)	545 (43•2%)
Fuel for cooking and heating (N=1264)				
Gas/electricity	225 (33.7%)	190 (37.9%)	35 (36.5%)	450 (35.6%)
Wood/coal	442 (66.3%)	311 (62.1%)	61 (63.5%)	814 (64.4%)
Housing type (N=1 264)				
Formal	400 (60•0%)	325 (64•9%)	59 (61•5%)	784 (62•0%)
Informal	267 (40•0%)	176 (35•1%)	37 (38•5%)	480 (38•0%)
Subdistrict-type (N=1 246)				
Ger	298 (45•1%)	231 (46•8%)	42 (46•2%)	571 (45•8%)
Apartment	107 (16•2%)	65 (13•2%)	14 (15•4%)	186 (14•9%)
Mixed	256 (38•7%)	198 (40•1%)	35 (38•5%)	489 (39•2%)
Breastfeeding (N=1 264)	392 (58•9%)	305 (60•8%)	41 (42•7%)	738 (58•4%)
Received antibiotics in 48 hours prior to admission (N=1 277)	330 (48•9%)	243 (48•0%)	44 (45•8%)	617 (48•3%)
Received hospital antibiotics prior to swab (N=1 239)	312 (48•0%)	239 (47•9%)	41 (45•6%)	592 (47•8%)
Pneumococcal carriage (N=1 283)	337 (49•8%)	232 (45•6%)	52 (53•6%)	621 (48•4%)
Percentage of lytA positive samples, serotype unknown (N=621)	45 (13•4%)	34 (14•7%)	10 (19•2%)	89 (14•3%)
PCV13 serotype carriage (N=1 194)	175 (27•7%)	73 (15•4%)	22 (25•3%)	270 (22•6%)

*Children are considered vaccinated if they have received at least two doses when administered at less than 12 months of age or at least one dose when administered at greater than or equal to 12 months of age.

† See Supplementary Figure 2 for histograms of age distribution by vaccination status

‡ Cold season refers to the winter months (Nov-March) and warm season refers to non-winter months.

§The definition of minimum living standard was changed by the Government of Mongolia between in 2017 from 170 000₮ to 241 000₮ per person/per month

Serotype unknown because the sample was culture negative or due to repeated technical difficulties with DNA extraction

**Table 2:** Characteristics of participants tested for pneumococcal carriage by subdistrict type, Ulaanbaatar, Mongolia, November 2015-March 2019

	Ger subdistrict	Mixed subdistrict	Apartment subdistrict	Total
	N=571	N=489	N=186	N=1246

Age (months) (N=1246)	14 (7-24)	18 (10-29)	15 (7-25)	15 (7-25)
Gender (N=1246)				
Female	257 (45.0%)	223 (45.6%)	72 (38.7%)	552 (44.3%)
Male	314 (55.0%)	266 (54.4%)	114 (61.3%)	694 (55.7%)
District (N=1246)				
Songinokhairkhan	360 (63.0%)	353 (72.2%)	64 (34.4%)	777 (62.4%)
Sukhbaatar	211 (37.0%)	136 (27.8%)	122 (65.6%)	469 (37.6%)
Season* (N=1246)				
Warm season	294 (51.5%)	247 (50.5%)	109 (58.6%)	650 (52.2%)
Cold season	277 (48.5%)	242 (49.5%)	77 (41.4%)	596 (47.8%)
Household income† (N=1174)				
At or below minimum level	281 (52.3%)	199 (43.2%)	64 (36.4%)	544 (46.3%)
Above minimum level	256 (47.7%)	262 (56.8%)	112 (63.6%)	630 (53.7%)
Maternal education (N=1229)				
Tertiary	246 (43.6%)	230 (47.6%)	152 (83.5%)	628 (51.1%)
Secondary school or less	318 (56.4%)	253 (52.4%)	30 (16.5%)	601 (48.9%)
Household crowding (people/room) (N=1214)				
≤ 3 people per room	267 (47.8%)	261 (54.8%)	140 (77.8%)	668 (55.0%)
> 3 people per room	291 (52.2%)	215 (45.2%)	40 (22.2%)	546 (45.0%)
Other children aged <5 years living in the house (N=1214)				
None	357 (64.1%)	347 (72.1%)	114 (64.8%)	818 (67.4%)
At least one child	200 (35.9%)	134 (27.9%)	62 (35.2%)	396 (32.6%)
Cigarette smoker in the house (N=1231)	309 (54.7%)	267 (55.2%)	122 (67.0%)	698 (56.7%)
Fuel for cooking and heating (N=1232)				
Gas/electricity	115 (20.4%)	161 (33.2%)	161 (88.5%)	437 (35.5%)
Wood/coal	450 (79.6%)	324 (66.8%)	21 (11.5%)	795 (64.5%)
Housing type (N=1232)				
Formal housing	308 (54.5%)	287 (59.2%)	172 (94.5%)	767 (62.3%)
Informal housing	257 (45.5%)	198 (40.8%)	10 (5.5%)	465 (37.7%)
Currently breastfeeding (N=1232)	335 (59.3%)	289 (59.6%)	93 (51.1%)	717 (58.2%)
Oral antibiotics within 48 hours prior to admission (N=1240)	264 (46.4%)	240 (49.4%)	91 (49.2%)	595 (48.0%)
Received hospital antibiotics prior to swab (N=1209)	280 (50.3%)	225 (47.3%)	74 (42.0%)	579 (47.9%)
PCV13 vaccination status‡ (N=1155)				
Under-vaccinated	298 (56.3%)	256 (56.4%)	107 (62.2%)	661 (57.2%)
Vaccinated	231 (43.7%)	198 (43.6%)	65 (37.8%)	494 (42.8%)
Pneumococcal carriage (N=1246)	287 (50.3%)	252 (51.5%)	64 (34.4%)	603 (48.4%)
lytA positive samples, serotype unknown§ (N=434)	52 (18.1%)	29 (11.5%)	7 (10.9%)	88 (14.6%)
Vaccine-type carriage (N=1158)	111 (21.4%)	116 (25.2%)	31 (17.3%)	258 (22.3%)

* Cold season refers to the winter months (Nov-March) and warm season refers to non-winter months.

† The definition of minimum living standard was changed by the Government of Mongolia between in 2017 from 170 000₮ to 241 000₮ per person/per month

‡ Children are considered vaccinated if they have received at least two doses when administered at less than 12 months of age or at least one dose when administered at greater than or equal to 12 months of age.

§ Serotype unknown because the sample was culture negative or due to repeated technical difficulties with DNA extraction

Out of 1 283 participants, the pneumococcal carriage rate was 48•4% (95% CI 45•6-51•2) and the majority were serotyped (85•7%). VT carriage rate was 22•6% (95% CI 20•3-25•1). Carriage characteristics were similar by vaccinations status, aside from lower VT carriage among vaccinated children (Table 1). Rate of AMR genes among lytA positive samples was 89•1% and the rate did not vary by vaccination status or over time (Supplementary Table 6).

### 3.2 Pneumococcal carriage over time

**Figure 1:**
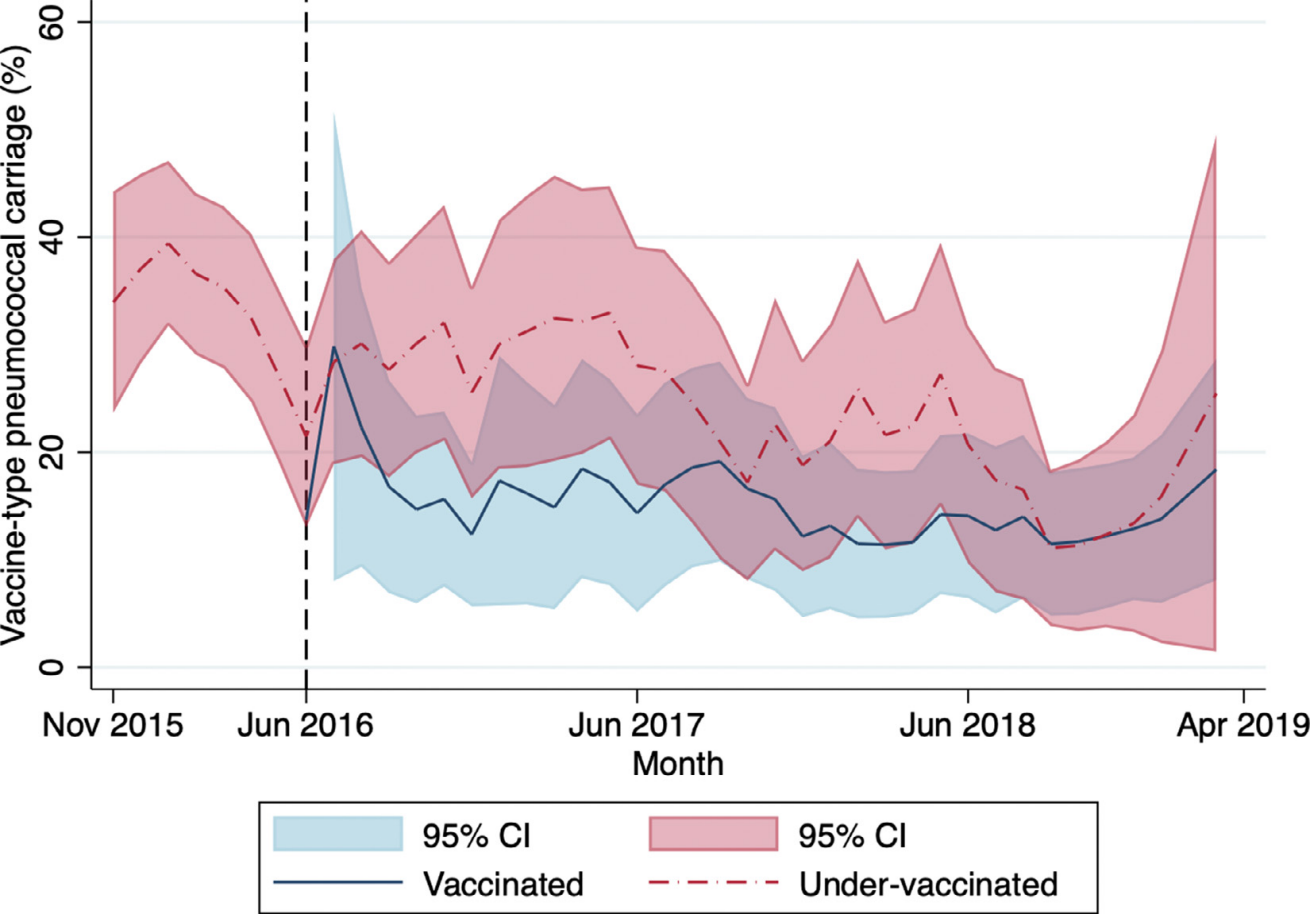
Monthly adjusted* prevalence of vaccine-type carriage (5-month rolling intervals) among 2–59-month-old participants with pneumonia, by PCV13 status, Ulaanbaatar, November 2015-March 2019; black dashed line (—) indicates date of PCV13 introduction *Adjusted by age group

VT carriage declined first among vaccinated participants, followed by under-vaccinated participants (Figure 1). There appeared to be a slight increase in VT carriage, especially among under-vaccinated participants, at the end of the study period. However case numbers were small since there were fewer forward months to include in the rolling five-month intervals. The individual serotypes identified are graphed by year in Supplementary Figure 2.

### 3.3 PCV13 coverage

**Figure 2:**
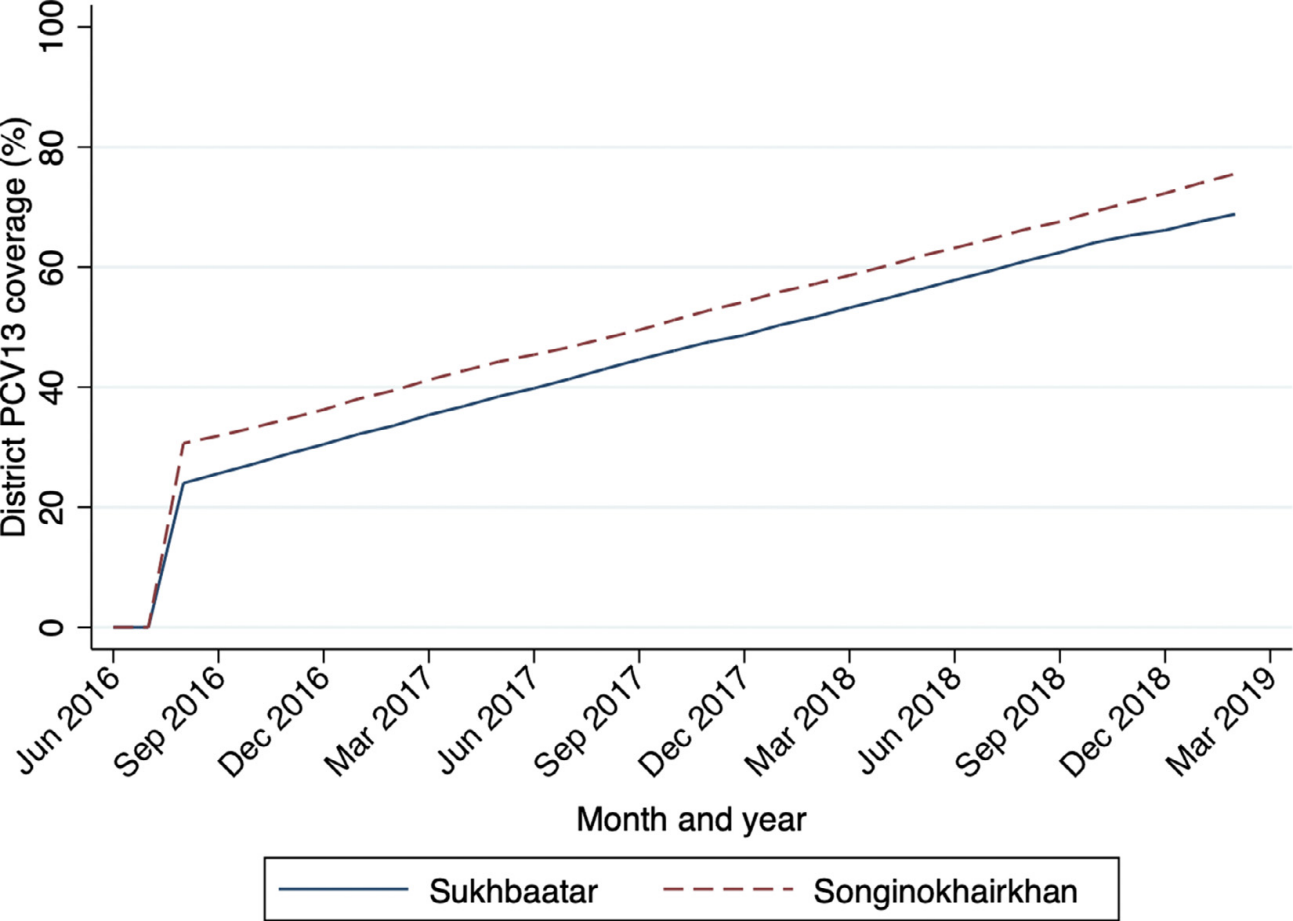
Monthly 13-valent pneumococcal conjugate vaccine (PCV13) coverage* among children under five years of age by district, Ulaanbaatar, 2016 – 2019 *Coverage defined as the number of children who had received at least two doses of PCV13 divided by the total number of children under five years within each district

Among children under five years of age, coverage of at least two doses of PCV13 increased to 70•2% in Sukhbaatar and 77•1% in Songinokhairkhan by March 2019 (Figure 2).

### 3.4 Association between PCV13 coverage and indirect effects

**Figure 3:**
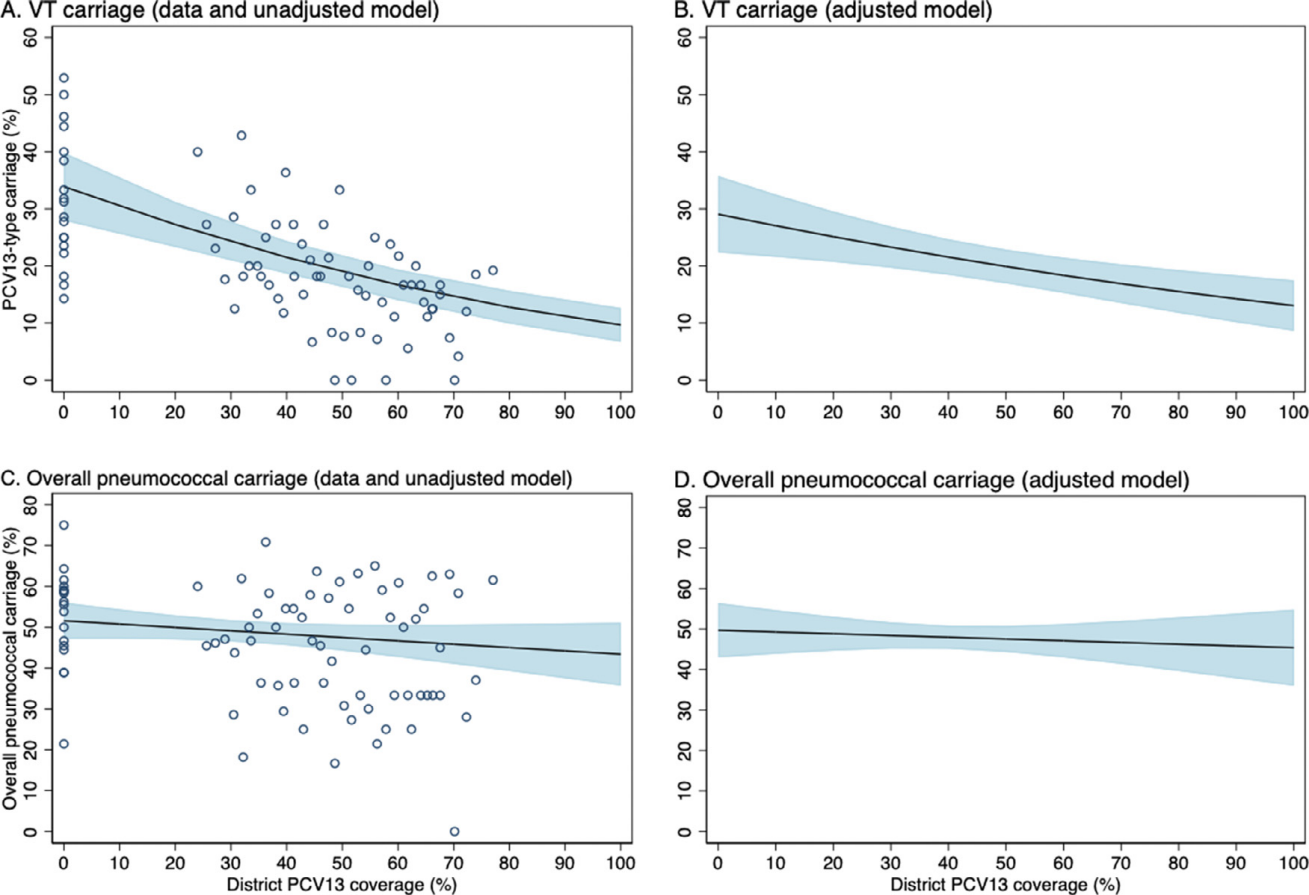
Adjusted* and unadjusted probability of vaccine-type (VT) and overall pneumococcal carriage by level of district 13-valent pneumococcal conjugate vaccine (PCV13) coverage†, Ulaanbaatar, Mongolia, 2015-2019; each circle represents data from children enrolled within the same month and district. *Adjusted by vaccination status, season, age group, subdistrict-type, housing-type, maternal education, household income, household crowding, number of children under five years of age, cigarette exposure, household fuel type, and antibiotic exposure † Coverage is defined as proportion of children under five years of age who have received at least two doses of PCV13

A description of the data available for this analysis are presented in Supplementary Table 7. For each increase in percentage point of PCV13 coverage, the adjusted odds of VT carriage decreased by 1•0% (0•4-1•7% [OR 0•990, 95% CI 0•983-0•996]; p=0•001) (Table 3). As coverage increases from 0% to 100%, the adjusted model predicts that PCV13 carriage will decrease from 29•1% (95% CI 22•4-35•8%) to 13•1% (95% CI 8•6-17•5%) (Figure 3). The adjusted PCV13 vaccine effectiveness against VT carriage was 39•1% (95% CI 11•4-58•1%, p=0•009). As expected, there was no evidence of an association between PCV13 coverage and overall pneumococcal carriage, the bias indicator, after adjustment (Table 3 and Figure 3).

**Table 3:** Adjusted* and unadjusted odds ratios of vaccine-type and overall pneumococcal carriage, by percentage increase in district 13-valent pneumococcal conjugate vaccine (PCV13) coverage† and individual PCV13 status‡, Ulaanbaatar, Mongolia, 2015-2019

	Unadjusted		Adjusted*	
	OR (95% CI)	p-value	OR (95% CI)	p-value

Vaccine-type carriage				
PCV13 coverage† at each participants’ resident district at time of enrolment	0·984 (0·980-0·989)	<0·001	0·990 (0·983-0·996)	0·001
Individual PCV13 status‡	0·494 (0·367-0·663)	<0·001	0·609 (0·419-0·886)	0·009
PCV13 effectiveness (%)§	50·6 (33·7-63·3)	<0·001	39·1 (11·4-58·1)	0·009
Overall pneumococcal carriage				
PCV13 coverage† at each participants’ resident district at time of enrolment	0·997 (0·992-1·001)	0·143	0·998 (0·992-1·005)	0·575
Individual PCV13 status‡	0·859 (0·712-1·04)	0·112	0·917 (0·749-1·124)	0·406
PCV13 effectiveness (%)§	14·1(-0·04-28·8)	0·112	8·3 (-12·4-25·1)	0·406

*Adjusted by PCV13 vaccination status, season, age group, subdistrict-type, housing type, maternal education, household income, household crowding, number of children under five years of age, cigarette exposure, household fuel type, and antibiotic exposure

†Coverage is defined as percentage of children under five years of age who have received at least two doses of PCV13.

‡Odds ratio of carriage among vaccinated compared to under-vaccinated participants, where vaccination is defined as receipt of at least two doses when administered at less than 12 months of age or at least one dose when administered at greater than or equal to 12 months of age.

§PCV13 effectiveness against carriage is calculated as one minus the odds ratio for the association between individual PCV13 status and carriage, multiplied by 100.

**Figure 4:**
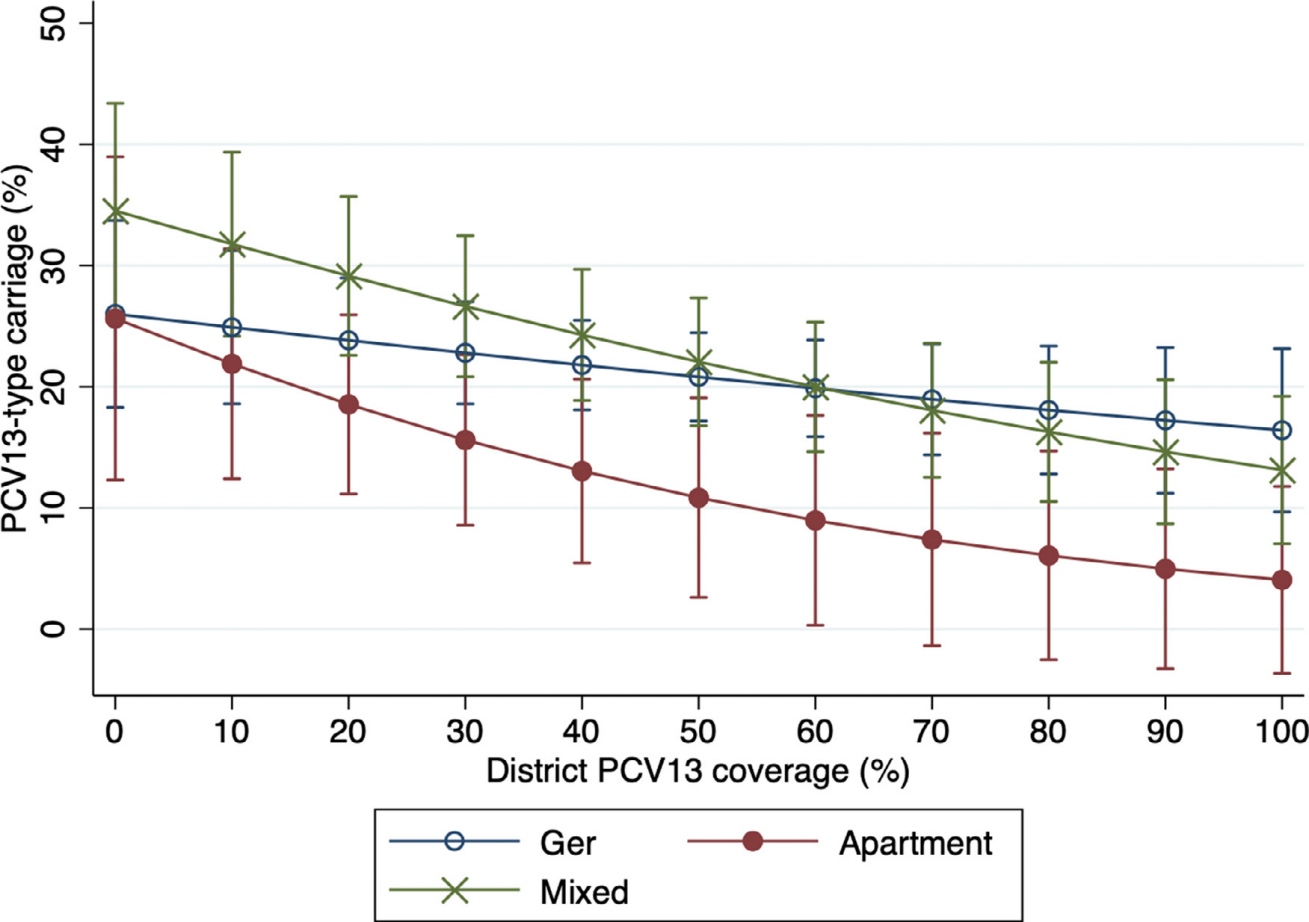
Adjusted* probability of vaccine-type carriage by level of 13-valent pneumococcal conjugate vaccine (PCV13) coverage† and subdistrict-type, Ulaanbaatar, Mongolia, 2015-2019 *Adjusted by vaccination status, season, age group, subdistrict-type, housing-type, maternal education, household income, household crowding, number of children under five years of age, cigarette exposure, household fuel type, and antibiotic exposure †Coverage is defined as proportion of children under five years of age who have received at least two doses of PCV13

While a Wald test did not indicate an interaction between coverage and subdistrict-type (p=0•100), there was a trend towards a steeper decline in the odds of VT carriage for each percentage point increase in PCV13 coverage among children from apartment subdistricts (1•5% [95% CI -0•7-3•7%]), followed by mixed housing subdistricts (0•7% [95% CI -0•3-1•7) and ger subdistricts (0•6% [95% CI -0•2-1•4%]) (Figure 4)

## 4 Discussion

Our study found evidence of indirect effects against VT carriage in Mongolia, including among children living in subdistricts comprised of informal (ger) housing, who have a higher burden of risk factors for pneumococcal disease. While coverage only reached 75% during our observation period, our model predicts that as coverage reaches 100% among children under five years of age, VT carriage would reduce by 55•0% (from 29•1% to 13•1% carriage rate) through indirect effects alone. Our results are consistent with community carriage surveys in Ulaanbaatar which found a 51% reduction in VT carriage in unvaccinated infants aged 5-8 weeks one year after PCV13 introduction.^20^ These reductions in VT carriage are expected to translate to reductions in VT disease, since carriage is a prerequisite for disease.^2^

We found that indirect effects against VT carriage started at low levels of vaccine coverage, challenging assumptions that high vaccine coverage is required for indirect effects. We recently reported similar associations between vaccine coverage and indirect effects against VT carriage in Lao Peoples’ Democratic Republic (Lao PDR) – where we predicted a 36% relative reduction in VT carriage as coverage increased to 60% despite heterogenous PCV13 coverage.^34^ This relationship has also been demonstrated for pneumococcal disease.^35^^,^^36^

While indirect effects were observed in children living in both formal and informal settlements, there was a trend towards greater reductions in VT carriage at lower levels of coverage for children living in apartment subdistricts, compared to children living in ger subdistricts. This may be attributable to the higher rate of VTs among children living in ger subdistricts.^20^ Children living in gers are at greater risk of pneumococcal infection due to household crowding – which contributes to increased exposure to both pneumococcus and viral co-infection.^37^ A lack of sanitation infrastructure and use of biomass fuels with resulting indoor air pollution may also contribute an increased risk of pneumococcal carriage.^38^ An alternative explanation is that our analysis, which was based on district-level coverage data, may have obscured differences in PCV coverage at the subdistrict level, with higher coverage resulting in greater reductions in VT carriage in apartment subdistricts. However previous reports indicate there is very little difference in vaccination coverage by wealth quintile within urban Ulaanbaatar. ^8^ Future studies designed to specifically study this may provide stronger evidence of interactions between PCV coverage and settlement-type.^39^

Our results are consistent with dynamic models suggesting that higher rates of PCV coverage are required to effectively interrupt person-to-person transmission in populations with high prevalence and transmission of VTs.^40^ Indeed, high vaccination coverage among children under five years alone may not be sufficient to control VTs in settings with high VT carriage prevalence. Studies from Fiji indicate that following PCV introduction, VT carriage was associated with physical contact with unvaccinated children 7-14 years of age.^41^ In these situations, alternative schedules or wider catch-up programs may be required to cover age groups acting as a reservoir for VTs.^42^

Our results also demonstrate strong direct effects against VT carriage for vaccinated children, in-line with previous estimates from other countries. In Lao PDR, which uses a schedule with three infant doses, we reported VE of 38•1% against VT carriage among children under five.^34^ In Brazil, three doses of PCV10 had 44% effectiveness against VT carriage among healthy children 7-11 months of age.^43^ Two PCV13 doses conferred 53% protection against VT carriage among healthy children aged ≤12 months in Israel.^26^ As expected, our estimates of VE against VT carriage are lower compared to effectiveness of PCV13 against VT disease.^44^

A key strength the study method pioneered by Ali et al ^45^ is the bias indicator analysis. Using similar methods, we showed no association between PCV13 coverage and overall pneumococcal carriage, which supports the internal validity of the study. Despite conducting this study among children with pneumonia, our results are consistent with previous pneumococcal carriage studies conducted among healthy children who had not been exposed to antibiotics. Furthermore, rates of VTs and AMR genes among our study population were similar to a previous community carriage survey of healthy children from Ulaanbaatar.^20^

An important limitation of our study was that estimates of vaccine coverage were based on administrative data.^46^ National estimates are likely to be reliable in Mongolia, where PCV13 is delivered through a well-structured public health care system and previous surveys indicate high accuracy of administrative coverage estimates, based on family health centre records.^17^^,^^47^ The electronic immunisation register, which was used as an adjunct to parent-held child health cards to determine participant vaccination status, has previously been demonstrated to have high degree of accuracy when compared to family health centre records.^23^ However, at a district level, population movement between districts eligible and ineligible for PCV means that the real vaccine coverage may be lower than the documented administrative estimates. This suggests that indirect effects may in fact be achievable in Mongolia at lower rates of vaccine coverage than we have reported.

Our definition of under-vaccinated children included those who received one dose of vaccine under 12 months of age and may benefit from some direct protection.^25^^,^^26^ Therefore, our estimates of VE may be lower than studies with comparisons against completely unvaccinated children, while overestimating the relationship between vaccine coverage and indirect effects. However, the effect is likely to be minimal since the majority of under-vaccinated children received no doses (81•2%).

Our findings have important policy implications for Mongolia and other countries in the region. Firstly, the cost-effectiveness of the vaccine greatly improves when the indirect effects are present and taken into account, which can help policy makers justify public funding of the vaccine.^48^ Secondly, indirect effects are necessary for the control of VT transmission, which is a prerequisite for the use of reduced dose schedules.^7^ Our results suggest VT control may not be achieved despite high coverage in the short-term in Mongolia. Further research is urgently needed to monitor long-term impacts and develop strategies to achieve VT control in high carriage settings, which would enable such settings to transition to cost-saving reduced dose schedules. We recommend prioritising research on understanding the remaining reservoirs for VT infection post-PCV and evaluating alternative vaccination strategies, such as additional booster doses or wider catch-up programs covering older children. While our study focuses on the direct and indirect effects of PCV, further research is also required to understand the degree to which corresponding increases in non-vaccine type pneumococcal carriage (also known as serotype replacement) may impact on the public health gains resulting from PCVs.^49^^,^^50^ Our results provide insight into the conditions necessary to generate strong indirect effects in Mongolia and have important implications for other countries in the region, many of which have yet to introduce PCV into routine childhood immunisation schedules.

## 5 Contributors

FMR conceived the idea and designed the study. TM, SLV, CVM, DN, EKM, JC and FMR supported the development of country-specific protocols. TM led study implementation with support from SLV, CVM, PB, MU, BS, DO, DL, DN, UC, EM, GD, EJ and AS. CS devised the microbiological approach and laboratory protocols with EMD and JH. Laboratory analysis was completed by CLP and MLN. JC, CN and FMR devised the analysis plan. JC completed the analysis and drafted the manuscript. All authors provided feedback to the draft manuscript and have approved the final version.

## Declaration of Competing Interest

KM, CDN ED, CS, TM and CVM are investigators on a separate study that received grant funding from Pfizer. JH is Co-founder and shareholder of BUGS Bioscience Ltd., a not-for-profit spin-out company of St George's, University of London. KF was on the National Foundation for Infectious Diseases Planning Committee for Vaccinology Conference.

## Appendix Supplementary materials

Image, application 1

Image, application 2

## References

[1] Wahl B, O'Brien KL, Greenbaum A (2018). Burden of Streptococcus pneumoniae and Haemophilus influenzae type b disease in children in the era of conjugate vaccines: global, regional, and national estimates for 2000–15. Lancet Glob Health.

[2] Simell B, Auranen K, Käyhty H (2012). The fundamental link between pneumococcal carriage and disease. Expert Rev Vaccines.

[3] Pilishvili T, Zell ER, Farley MM (2010). Risk factors for invasive pneumococcal disease in children in the era of conjugate vaccine use. Pediatrics.

[4] Mullholland K, Weber MW. (2016). Pneumonia in Children: Epidemiology, Prevention and Treatment.

[5] Feikin DR, Kagucia EW, Loo JD (2013). Serotype-specific changes in invasive pneumococcal disease after pneumococcal conjugate vaccine introduction: a pooled analysis of multiple surveillance sites. PLOS Med.

[6] Davis SM, Deloria-Knoll M, Kassa HT, O'Brien KL (2013). Impact of pneumococcal conjugate vaccines on nasopharyngeal carriage and invasive disease among unvaccinated people: Review of evidence on indirect effects. Vaccine.

[7] Flasche S, Van Hoek AJ, Goldblatt D (2015). The Potential for Reducing the Number of Pneumococcal Conjugate Vaccine Doses While Sustaining Herd Immunity in High-Income Countries. PLOS Med.

[8] Center for Health Development. Health Indicators 2016 - Mongolia. Ulaanbaatar, Mongolia https://untobaccocontrol.org/impldb/wp-content/uploads/mongolia_2018_annex-1_health_indicator_2016.pdf (accessed Jan 19, 2021).

[9] von Mollendorf C, La Vincente S, Ulziibayar M (2019). Epidemiology of pneumonia in the pre-pneumococcal conjugate vaccine era in children 2-59 months of age, in Ulaanbaatar, Mongolia, 2015-2016. PLOS One.

[10] Jadambaa A, Spickett J, Badrakh B, Norman RE. (2014). The Impact of the Environment on Health in Mongolia: A Systematic Review. Asia Pac J Public Health.

[11] National Statistics Office of Mongolia. 2015 Population and Housing By-Census of Mongolia: National Report. 2016 https://www.en.nso.mn:443/content/166 (accessed Sept 27, 2020).

[12] Fan P, Chen J, John R. (2016). Urbanization and environmental change during the economic transition on the Mongolian Plateau: Hohhot and Ulaanbaatar. Environ Res.

[13] Park H, Fan P, John R, Ouyang Z, Chen J. (2019). Spatiotemporal changes of informal settlements: Ger districts in Ulaanbaatar, Mongolia. Landsc Urban Plan.

[14] von Mollendorf C, Cohen C, de Gouveia L (2015). Risk Factors for Invasive Pneumococcal Disease Among Children Less Than 5 Years of Age in a High HIV Prevalence Setting, South Africa, 2010 to 2012. Pediatr Infect Dis J.

[15] Lancet T. (2017). Urbanisation, inequality, and health in Asia and the Pacific. The Lancet.

[16] International Vaccine Access Centre. ViewHub. https://view-hub.org/(accessed Jan 11, 2021).

[17] La Vincente SF, Von Mollendorf C, Ulziibayar M (2019). Evaluation of a phased pneumococcal conjugate vaccine introduction in Mongolia using enhanced pneumonia surveillance and community carriage surveys: A study protocol for a prospective observational study and lessons learned. BMC Public Health.

[18] Chan J, Nguyen CD, Lai JYR (2018). Determining the pneumococcal conjugate vaccine coverage required for indirect protection against vaccine-type pneumococcal carriage in low and middle-income countries: a protocol for a prospective observational study. BMJ Open.

[19] World Health Organization. Pneumococcus: Vaccine Preventable Diseases Surveillance Standards. 2018 https://www.who.int/publications/m/item/vaccine-preventable-diseases-surveillance-standards-pneumococcus (accessed Jan 5, 2021).

[20] von Mollendorf C, Dunne EM, La Vincente S (2019). Pneumococcal carriage in children in Ulaanbaatar, Mongolia before and one year after the introduction of the 13-valent pneumococcal conjugate vaccine. Vaccine.

[21] Ali M, Emch M, von Seidlein L (2005). Herd immunity conferred by killed oral cholera vaccines in Bangladesh: a reanalysis. The Lancet.

[22] World Health Organization and UNICEF. Mongolia: WHO and UNICEF estimates of immunization coverage: 2019 revision. 2020 http://158.232.12.119/immunization/monitoring_surveillance/data/mng.pdf (accessed Oct 25, 2020).

[23] Chan J, Mungun T, Dorj N (2017). High agreement between the new Mongolian electronic immunization register and written immunization records: a health centre based audit. West Pac Surveill Response J WPSAR.

[24] Satzke C, Turner P, Virolainen-Julkunen A (2013). Standard method for detecting upper respiratory carriage of Streptococcus pneumoniae: updated recommendations from the World Health Organization Pneumococcal Carriage Working Group. Vaccine.

[25] Scott P, Rutjes AWS, Bermetz L (2011). Comparing pneumococcal conjugate vaccine schedules based on 3 and 2 primary doses: systematic review and meta-analysis. Vaccine.

[26] Lewnard JA, Givon-Lavi N, Dagan R. (2020). Dose-specific Effectiveness of 7- and 13-Valent Pneumococcal Conjugate Vaccines Against Vaccine-serotype Streptococcus pneumoniae Colonization in Children. Clin Infect Dis Off Publ Infect Dis Soc Am.

[27] Satzke C, Dunne EM, Choummanivong M, et al. Pneumococcal carriage in vaccine-eligible children and unvaccinated infants in Lao PDR two years following the introduction of the 13-valent pneumococcal conjugate vaccine. 2018. DOI:10.1016/j.vaccine.2018.10.077. 10.1016/j.vaccine.2018.10.077 30502068

[28] Newton R, Hinds J, Wernisch L. (2011). Empirical Bayesian models for analysing molecular serotyping microarrays. BMC Bioinformatics.

[29] Satzke C, Dunne EM, Porter BD, Klugman KP, Mulholland EK (2015). The PneuCarriage Project: A Multi-Centre Comparative Study to Identify the Best Serotyping Methods for Examining Pneumococcal Carriage in Vaccine Evaluation Studies. PLOS Med.

[30] Ahmad OB, Boschi-Pinto C, Lopez AD, Murray CJ, Lozano R, Inoue M. Age Standardization of Rates: A new WHO Standard. World Health Organization.

[31] Deen J, Ali M, Sack D. (2014). Methods to Assess the Impact of Mass Oral Cholera Vaccination Campaigns under Real Field Conditions. PLOS One.

[32] StataCorp. Stata Statistical Software: Release 15. 2019.

[33] Baselinetable Donath S. (2018). A command for creating oneand two-way tables of summary statistics. Stata J.

[34] Chan J, Lai JY, von Mollendorf C (2019). Determining the pneumococcal conjugate vaccine coverage required for indirect protection within Asia and the Pacific: a prospective observational study. Lancet Glob Health.

[35] Shiri T, Datta S, Madan J (2017). Indirect effects of childhood pneumococcal conjugate vaccination on invasive pneumococcal disease: a systematic review and meta-analysis. Lancet Glob Health.

[36] Chan J, Gidding HF, Blyth C (2018). Determining the vaccination coverage required for indirect protection against invasive pneumococcal disease, Australia. National Immunisation Conference.

[37] Collins DA, Hoskins A, Snelling T (2017). Predictors of pneumococcal carriage and the effect of the 13-valent pneumococcal conjugate vaccination in the Western Australian Aboriginal population. Pneumonia Nathan Qld.

[38] Dunne EM, Choummanivong M, Neal EFG (2019). Factors associated with pneumococcal carriage and density in infants and young children in Laos PDR. PLOS One.

[39] Brookes ST, Whitely E, Egger M, Smith GD, Mulheran PA, Peters TJ. (2004). Subgroup analyses in randomized trials: risks of subgroup-specific analyses;: power and sample size for the interaction test. J Clin Epidemiol.

[40] Lourenço J, Obolski U, Swarthout TD (2019). Determinants of high residual post-PCV13 pneumococcal vaccine-type carriage in Blantyre, Malawi: a modelling study. BMC Med.

[41] Neal EFG, Flasche S, Nguyen CD (2020). Associations between ethnicity, social contact, and pneumococcal carriage three years post-PCV10 in Fiji. Vaccine.

[42] Swarthout TD, Fronterre C, Lourenço J (2020). High residual carriage of vaccine-serotype Streptococcus pneumoniae after introduction of pneumococcal conjugate vaccine in Malawi. Nat Commun.

[43] Andrade AL, Ternes YM, Vieira MA (2014). Direct effect of 10-valent conjugate pneumococcal vaccination on pneumococcal carriage in children Brazil. PLOS One.

[44] Berman-Rosa M, O'Donnell S, Barker M, Quach C (2020). Efficacy and Effectiveness of the PCV-10 and PCV-13 Vaccines Against Invasive Pneumococcal Disease. Pediatrics.

[45] Ali M, Emch M, von Seidlein L (2005). Herd immunity conferred by killed oral cholera vaccines in Bangladesh: a reanalysis. The Lancet.

[46] Cutts FT, Claquin P, Danovaro-Holliday MC, DA Rhoda (2016). Monitoring vaccination coverage: Defining the role of surveys. Vaccine.

[47] Immunization country profiles: Mongolia. UNICEF DATA. 2018; published online July 17. https://data.unicef.org/resources/immunization-country-profiles/(accessed Nov 24, 2020).

[48] Holubar M, Stavroulakis MC, Maldonado Y, Ioannidis JPA, Contopoulos-Ioannidis D (2017). Impact of vaccine herd-protection effects in cost-effectiveness analyses of childhood vaccinations. A quantitative comparative analysis. PLOS One.

[49] Chan J, Nguyen CD, Dunne EM (2019). Using pneumococcal carriage studies to monitor vaccine impact in low- and middle-income countries. Vaccine.

[50] Lewnard JA, Hanage WP. (2019). Making sense of differences in pneumococcal serotype replacement. Lancet Infect Dis.

