# Supplementary material for: Direct and indirect effects of 13-valent pneumococcal conjugate vaccine on pneumococcal carriage in children hospitalised with pneumonia from formal and informal settlements in Mongolia: an observational study
Source: Lancet Reg Health West Pac. 2021 Jul 30;15:100231. doi: 10.1016/j.lanwpc.2021.100231 (PMC8342962; doi:10.1016/j.lanwpc.2021.100231)
Supplement: Supplementary file 1 [file mmc1.pdf]

Supplementary appendix

Vaccination coverage – data collection and calculation

In Mongolia, pneumococcal conjugate vaccine (PCV) is delivered through a well-structured publicly-funded health care system. (1) Within Sukhbaatar and Songinokhairkhan districts, vaccinations are administered within one of 19 Family Health Centers (FHC).(2) Each child is registered to a particular FHC based on their location of residence. District hospitals notify local FHCs of births for follow-up of infant and childhood health care. Health staff document vaccinations both in parent-held records and in written or electronic registration records within FHCs. Registration books document the following minimum information: name, national identification (ID) number (unique identification number given at birth), age, address, phone number and dates of vaccine administration. As such, the vast majority of children eligible for vaccinations and vaccination doses administered will be captured within FHC records. The main source of inaccuracy relates to delay in registering and transfer of records when families move between subdistricts. However, there are incentives for families to register with their FHCs in order to access health care and other social services.

Expanded Program of Immunizations (EPI) staff collated data on monthly number of children eligible and vaccinated for each PCV dose from FHC records specifically for the purposes of this study. Data were collected for both the routine immunisation program and the limited-time supplementary catch-up program that coincided with the introduction of the PCV, which covered children up to 23 months of age. The raw data for each district are presented in supplementary tables 1 and 2.

For routine monitoring, vaccination coverage is calculated as the number of age-eligible children who had received at least two doses divided by total number of age-eligible children. At a national level, Mongolia routinely achieves very high coverage of routine immunisations (greater than 95% coverage). Over the past 10 years, official estimates based on administrative data are consistent with population-based household surveys from 2009, 2011, 2016 and 2017. (3)

For the purposes of this study, we calculated monthly vaccination coverage as the number of children who had received at least two doses of the 13-valent PCV (PCV13) divided by total number of children under five years within each district. Population data estimates for children under five years of age was provided by the Mongolian Department of Health for 2018.

Supplementary Table 1: Number of vaccine-eligible and vaccinated children by month, dose and program, Sukhbaatar, Mongolia, 2016-2019

	Routine immunisation									Supplementary program			
	Dose 1			Dose 2			Dose 3			Dose 1		Dose 2	
	No. eligible (2-3 months)	No. eligible vaccinated	No. vaccinated (delayed*)	No. eligible (4-5 months)	No. eligible vaccinated	No. vaccinated (delayed*)	No. eligible (9-10 months)	No. eligible vaccinated	No. vaccinated (delayed*)	No. eligible (3-23 months)	No. eligible vaccinated	No. eligible (5-23 months)	No. eligible vaccinated
Jun-16	263	253	-	-	-	-	-	-	-	4163	3858	-	-
Jul-16	281	271	3	-	-	-	-	-	-	-	-	-	-
Aug-16	276	266	0	253	229	0	-	-	-	-	-	3858	3172
Sep-16	299	289	3	271	237	3	-	-	-	-	-	-	-
Oct-16	274	264	1	266	243	5	-	-	-	-	-	-	-
Nov-16	245	235	0	289	248	6	-	-	-	-	-	-	-
Dec-16	236	226	6	264	223	3	-	-	-	-	-	-	-
Jan-17	255	254	0	293	285	0	194	182	1	-	-	-	-
Feb-17	210	208	1	239	238	0	212	201	7	-	-	-	-
Mar-17	257	256	2	304	296	4	283	258	8	-	-	-	-
Apr-17	190	189	3	245	236	2	290	268	4	-	-	-	-
May-17	245	241	0	279	271	1	297	286	8	-	-	-	-
Jun-17	231	230	3	231	230	0	340	284	2	-	-	-	-
Jul-17	233	230	0	260	218	3	262	251	1	-	-	-	-
Aug-17	248	244	0	281	246	2	332	328	0	-	-	-	-

Sep-17	269	268	0	263	256	8	260	255	13	-	-	-	-
Oct-17	280	280	2	252	247	2	246	241	10	-	-	-	-
Nov-17	244	243	0	251	248	0	177	176	3	-	-	-	-
Dec-17	234	233	0	190	190	0	126	125	1	-	-	-	-
Jan-18	248	248	0	285	284	0	268	268	0	-	-	-	-
Feb-18	202	201	0	228	227	0	255	254	2	-	-	-	-
Mar-18	250	250	0	270	269	0	287	286	3	-	-	-	-
Apr-18	213	213	0	251	250	0	285	285	1	-	-	-	-
May-18	245	245	1	265	265	2	277	276	2	-	-	-	-
Jun-18	261	261	1	271	271	0	250	249	4	-	-	-	-
Jul-18	236	236	0	256	256	1	241	241	1	-	-	-	-
Aug-18	280	280	2	279	278	3	230	228	2	-	-	-	-
Sep-18	224	224	3	236	236	10	200	197	4	-	-	-	-
Oct-18	299	299	3	282	282	3	256	256	3	-	-	-	-
Nov-18	235	235	5	200	200	2	189	188	2	-	-	-	-
Dec-18	184	184	6	143	143	5	128	127	4	-	-	-	-
Jan-19	239	238	2	246	235	0	275	263	2	-	-	-	-
Feb-19	203	200	1	207	205	6	245	238	2	-	-	-	-
Mar-19	222	222	0	229	226	2	259	255	2	-	-	-	-
Apr-19	247	246	2	242	203	4	246	230	0	-	-	-	-
May-19	218	210	0	245	240	2	227	219	1	-	-	-	-
Jun-19	287	277	3	274	258	3	247	235	0	-	-	-	-

*Delayed vaccination is defined as a vaccine administered over one month past the scheduled date

Supplementary Table 2: Number of vaccine-eligible and vaccinated children by month, dose and program, Songinokhairkhan, Mongolia, June 2016 – June 2019

	Routine immunisation (<1yo)									Supplementary program			
	Dose 1			Dose 2			Dose 3			Dose 1		Dose 2	
	No. eligible (2-3 months)	No. eligible vaccinated	No. vaccinated (delayed*)	No. eligible (4-5 months)	No. eligible vaccinated	No. vaccinated (delayed*)	No. eligible (9-10 months)	No. eligible vaccinated	No. vaccinated (delayed*)	No. eligible (3-23 months)	No. eligible vaccinated	No. eligible (5-23 months)	No. eligible vaccinated
Jun-16	644	644	-	-	-	-	-	-	-	12925	12292	-	-
Jul-16	378	373	0	-	-	-	-	-	-	-	-	-	-
Aug-16	692	687	1	551	549		-	-	-	-	-	12292	11055
Sep-16	624	618	2	516	506	1	-	-	-	-	-	-	-
Oct-16	696	685	2	570	566	1	-	-	-	-	-	-	-
Nov-16	743	738	1	631	618		-	-	-	-	-	-	-
Dec-16	640	616	2	622	605		-	-	-	-	-	-	-
Jan-17	745	728	29	734	728	30	481	460		-	-	-	-
Feb-17	481	458	17	533	514	42	431	406	6	-	-	-	-
Mar-17	629	620	19	681	666	62	683	658	31	-	-	-	-
Apr-17	483	458	21	605	554	40	625	587	26	-	-	-	-
May-17	586	565	7	570	533	48	666	643	12	-	-	-	-
Jun-17	480	470	7	382	365	110	615	592	26	-	-	-	-
Jul-17	440	434	168	486	463	26	566	534	11	-	-	-	-
Aug-17	659	651	12	577	566	45	622	599	27	-	-	-	-
Sep-17	644	636	16	562	538	19	556	515	34	-	-	-	-
Oct-17	671	661	10	651	633	52	480	464	44	-	-	-	-
Nov-17	650	640	10	643	616	40	427	406	45	-	-	-	-

Dec-17	386	379	148	552	527	28	385	357	27	-	-	-	-
Jan-18	693	681	14	625	605	69	631	615	61	-	-	-	-
Feb-18	547	542	12	496	481	47	445	430	18	-	-	-	-
Mar-18	675	662	17	603	573	12	657	629	45	-	-	-	-
Apr-18	576	569	16	576	554	49	600	574	41	-	-	-	-
May-18	662	651	15	570	554	123	595	571	41	-	-	-	-
Jun-18	647	624	19	560	516	40	543	509	24	-	-	-	-
Jul-18	626	620	11	573	546	32	480	460	32	-	-	-	-
Aug-18	672	669	17	649	624	27	582	563	24	-	-	-	-
Sep-18	577	568	8	534	502	9	484	464	23	-	-	-	-
Oct-18	729	722	21	666	644	51	629	602	42	-	-	-	-
Nov-18	678	674	12	598	571	54	470	453	49	-	-	-	-
Dec-18	622	610	11	564	535	48	492	468	26	-	-	-	-
Jan-19	648	642	25	668	643	41	578	554	38	-	-	-	-
Feb-19	567	554	29	607	576	49	536	503	42	-	-	-	-
Mar-19	618	613	19	597	565	40	614	577	36	-	-	-	-
Apr-19	640	632	27	609	582	53	590	574	49	-	-	-	-
May-19	648	642	22	585	557	63	573	552	39	-	-	-	-
Jun-19	616	610	16	543	512	51	491	477	26	-	-	-	-

*Delayed vaccination is defined as a vaccine administered over one month past the scheduled date

Variable definition and selection

Supplementary Table 3: Description of study variables

**DAG variables**	**Study variables**	**Categories**	**Description**
Age	Age group	2-11 months, 12-23 months, 24-59 months	Categorical
Gender	Gender	Male and female	Binary
Season	Season	Cold season (November- March, inclusive) and warm season (April-October, inclusive)	Binary
Crowding	Crowding	Less than or equal to 3 people per room, greater than 3 people per room	Binary, based on number of people per room
Other children (<5 years) in the house	Other children (<5 years) in the house	No other children under five years of age in the house and at least one other child under five years of age in the house	Binary
Smoker in the house	Smoker in the house	No smokers in the house and at least one smoker in the house	Binary
Fuel type	Fuel for cooking and heating	Uses gas/electricity for cooking, uses wood/coal for cooking	Binary, classified according to fuels associated with indoor air pollution
Housing type	Housing type	Formal and informal	Binary, informal housing refers to gers while formal housing refers to apartments and houses
Subdistrict type	Subdistrict type	Ger, apartment or mixed	Categorical
Socioeconomic status	Household income	At or below minimum, above minimum	Binary, the definition of minimum living standard was changed by the Government of Mongolia between 2015 (170,000₮ per person/per month) and 2017 (241,000₮ per person/per month).
	Mother's education	Secondary school or less, Tertiary	Binary
Breastfeeding	Breastfeeding	Yes, No	Binary, currently being breastfed or not
Antibiotic exposure	Pre-admission antibiotics	Yes, No	Binary, self-reported
	Hospital antibiotics administered prior to swab	Yes, No	Binary, based on hospital records

	Any antibiotics	Yes, No	Binary, any receipt of antibiotic either pre-admission or in hospital
Vaccination status	Vaccination status	Vaccinated, under-vaccinated	Binary

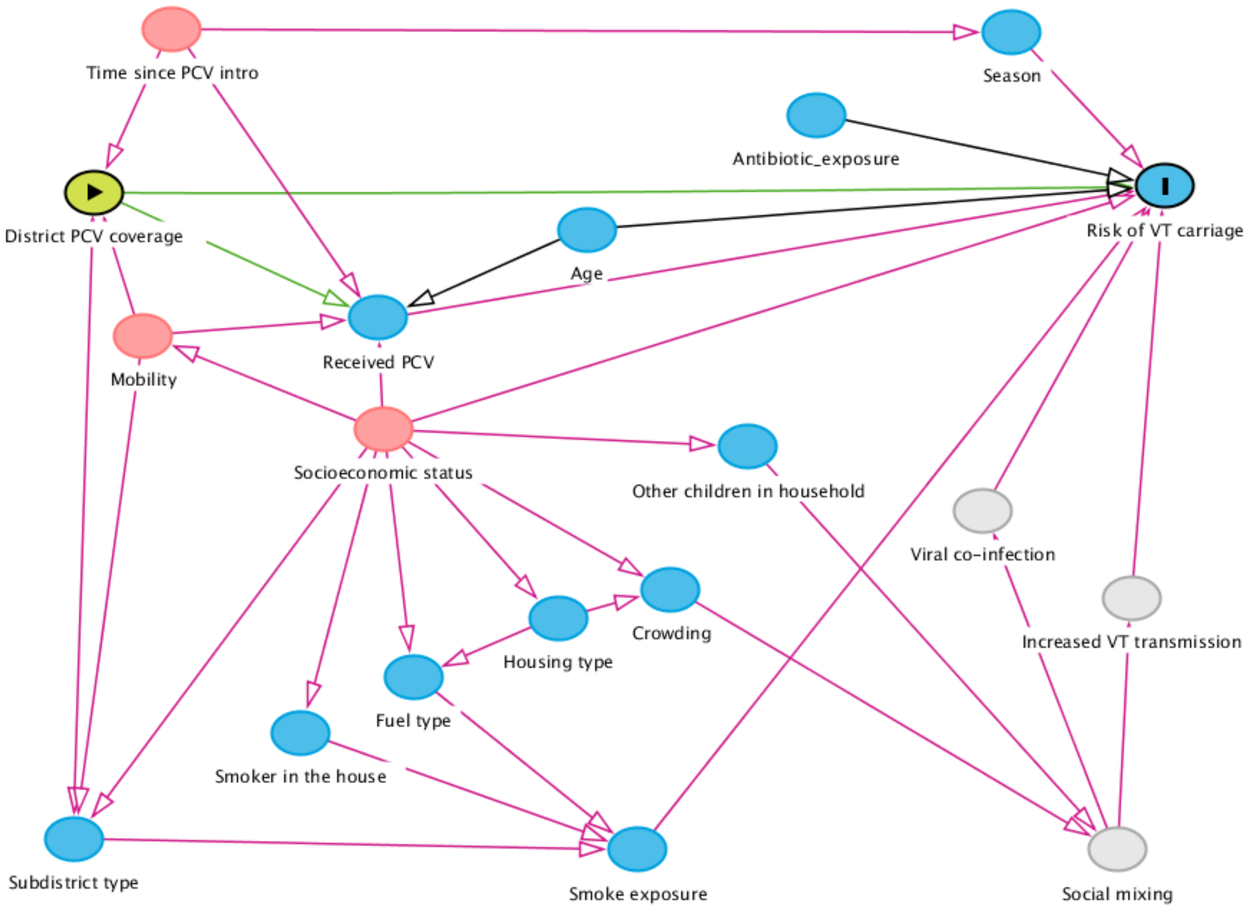

Supplementary figure 1: Directed acyclic graph of the association between district-level PCV13 (exposure) and risk of VT carriage (outcome); socioeconomic status was indicated by household income and maternal education in the data

We developed this directed acyclic graph (DAG)(4) to assist with the identification of potential confounders. Arrows are used to indicate causal pathways between exposure and outcome. The green lines highlight the causal relationship under investigation and the pink lines highlight potential biasing pathways. The blue variables are ancestors of the outcome, i.e. they directly or indirectly cause the outcome. Red variables are ancestors of both exposure and outcome. Grey variables represent unobserved variables.

Mongolia has a comprehensive childhood immunisation and primary health care program for children. It routinely achieves high rates of vaccination coverage. The key risk factors for under-

vaccination of PCV relate to high rates of population mobility. During this phased introduction, PCV was only available within certain districts in Ulaanbaatar. Rural to urban migration, seasonal migration and movement within Ulaanbaatar (particularly associated with informal housing and rental apartments) means that children may be under-vaccinated. Note that this diagram includes two unmeasured variables: mobility and social mixing.

Based on this diagram, we identified that adjusting for vaccination status, season, age group, subdistrict-type, housing-type, maternal education, household income, household crowding, number of children under five years of age, cigarette exposure, household fuel type, and antibiotic exposure would block biasing pathways.

Supplementary Table 4: Characteristics of participants by testing status, Ulaanbaatar, Mongolia, November 2015-March 2019

	Carriage data not available (N=4766)	Carriage data available (N=1283)	Total (N=6049)
Age (months) (N=6046)	16 (8-27)	15 (7-25)	16 (8-27)
Gender (N=6044)			
Female	2299 (48.3%)	569 (44.3%)	2868 (47.5%)
Male	2462 (51.7%)	714 (55.7%)	3176 (52.5%)
Season* (N=6046)			
Warm season	1154 (24.2%)	674 (52.5%)	1828 (30.2%)
Cold season	3609 (75.8%)	609 (47.5%)	4218 (69.8%)
Household income† (N=5406)			
At or below minimum	2010 (47.9%)	559 (46.4%)	2569 (47.5%)
Above minimum	2190 (52.1%)	647 (53.6%)	2837 (52.5%)
Maternal education (N=5622)			
Tertiary	2252 (51.6%)	641 (50.8%)	2893 (51.5%)
Secondary school or less	2109 (48.4%)	620 (49.2%)	2729 (48.5%)
Crowding (people/room) (N=5563)			
<=3 people per room	2360 (54.6%)	682 (54.8%)	3042 (54.7%)
> 3 people per room	1959 (45.4%)	562 (45.2%)	2521 (45.3%)
Other children aged <5 years in the house (N=5590)			
None	2877 (66.2%)	838 (67.3%)	3715 (66.5%)
At least one child	1468 (33.8%)	407 (32.7%)	1875 (33.5%)
Smoker in the house (N=5636)	1916 (43.8%)	545 (43.2%)	2461 (43.7%)
Housing type (N=5641)			
Formal housing	2721 (62.2%)	784 (62.0%)	3505 (62.1%)
Informal housing	1656 (37.8%)	480 (38.0%)	2136 (37.9%)
Subdistrict type (N=5859)			
Ger	2091 (45.3%)	571 (45.8%)	2662 (45.4%)
Apartment	729 (15.8%)	186 (14.9%)	915 (15.6%)
Mixed	1793 (38.9%)	489 (39.2%)	2282 (38.9%)
Breastfeeding (N=5641)	2425 (55.4%)	738 (58.4%)	3163 (56.1%)

Received antibiotics in 48 hours prior to admission (N=6010)	2372 (50.1%)	617 (48.3%)	2989 (49.7%)
Received hospital antibiotics prior to swab (N=5847)	2145 (46.5%)	592 (47.8%)	2737 (46.8%)
Vaccination status‡ (N=5076)			
Under-vaccinated	2630 (59.2%)	677 (57.1%)	3307 (58.7%)
Vaccinated	1815 (40.8%)	509 (42.9%)	2324 (41.3%)

* Cold season refers to the winter months (Nov-March) and warm season refers to non-winter months.
†The definition of minimum living standard was changed by the Government of Mongolia between in 2017 from 170,000₮ to 241,000₮ per person/per month
‡ Children are considered vaccinated if they have received at least two doses when administered at less than 12 months of age or at least one dose when administered at greater than or equal to 12 months of age.

Supplementary figure 2: Age distribution of participants that were tested for pneumococcal carriage by vaccination status, Ulaanbaatar, Mongolia, November 2015-March 2019

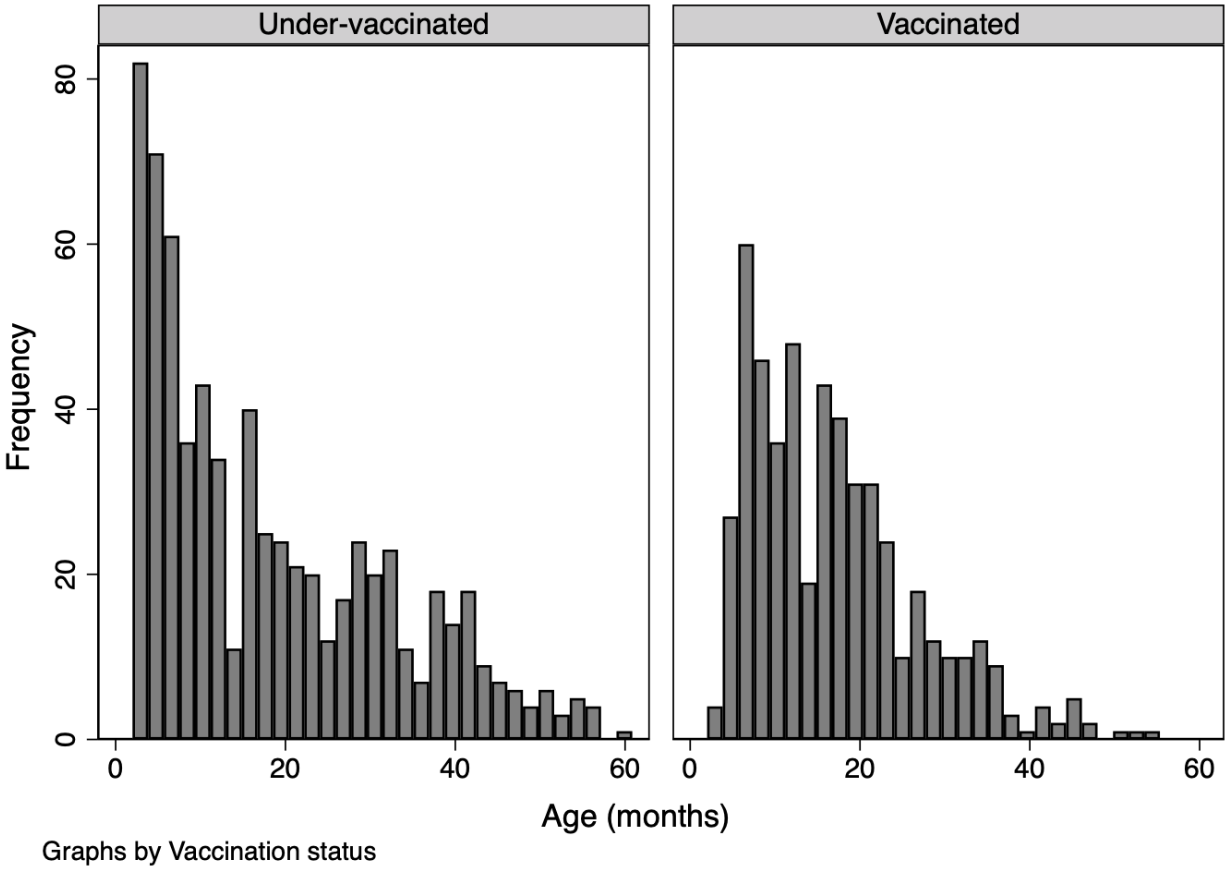

Supplementary Table 5: Characteristics of participants that were tested for pneumococcal carriage by year of enrolment, Ulaanbaatar, Mongolia, November 2015-March 2019

	Pre-PCV (Nov 2015 - May 2016)	Year 1 post-PCV (Jun 2016 - May 2017)	Year 2 post-PCV (Jun 2017 - May 2018)	Year 3 post-PCV (Jun 2018 – May 2019)	Total (Nov 2015 - Mar 2019)
	N=220	N=387	N=394	N=282	N=1283
Age (months) (N=1283) †	15 (7-23)	14 (7-24)	17 (9-27)	14 (6-25)	15 (7-25)
Gender (N=1283)					
Female	101 (45·9%)	183 (47·3%)	162 (41·1%)	123 (43·6%)	569 (44·3%)
Male	119 (54·1%)	204 (52·7%)	232 (58·9%)	159 (56·4%)	714 (55·7%)
District (N=1283)					
Songinokhairkhan	111 (50·5%)	235 (60·7%)	235 (59·6%)	220 (78·0%)	801 (62·4%)
Sukhbaatar	109 (49·5%)	152 (39·3%)	159 (40·4%)	62 (22·0%)	482 (37·6%)
Season* (N=1283)					
Warm season	64 (29·1%)	226 (58·4%)	229 (58·1%)	155 (55·0%)	674 (52·5%)
Cold season	156 (70·9%)	161 (41·6%)	165 (41·9%)	127 (45·0%)	609 (47·5%)
Household income† (N=1206)					
At or below minimum level	93 (46·0%)	174 (49·2%)	182 (48·8%)	110 (39·7%)	559 (46·4%)
Above minimum level	109 (54·0%)	180 (50·8%)	191 (51·2%)	167 (60·3%)	647 (53·6%)
Maternal education (N=1261)					
Tertiary	107 (49·1%)	186 (50·0%)	211 (54·0%)	137 (48·9%)	641 (50·8%)
Secondary school or less	111 (50·9%)	186 (50·0%)	180 (46·0%)	143 (51·1%)	620 (49·2%)
Household crowding (people/room) (N=1244)					
≤ 3 people per room	124 (57·7%)	172 (47·5%)	237 (60·6%)	149 (54·0%)	682 (54·8%)
> 3 people per room	91 (42·3%)	190 (52·5%)	154 (39·4%)	127 (46·0%)	562 (45·2%)
Other children aged <5 years living in the house (N=1245)					
None	141 (64·7%)	229 (64·1%)	280 (71·4%)	188 (67·6%)	838 (67·3%)
At least one child	77 (35·3%)	128 (35·9%)	112 (28·6%)	90 (32·4%)	407 (32·7%)
Cigarette smoker in the house (N=1263)	95 (43·6%)	182 (48·9%)	151 (38·4%)	117 (41·8%)	545 (43·2%)
Fuel for cooking and heating (N=1264)					

Gas/electricity	67 (30.7%)	119 (31.9%)	163 (41.5%)	101 (36.1%)	450 (35.6%)
Wood/coal	151 (69.3%)	254 (68.1%)	230 (58.5%)	179 (63.9%)	814 (64.4%)
Housing type (N=1264)					
Formal housing	129 (59·2%)	223 (59·8%)	255 (64·9%)	177 (63·2%)	784 (62·0%)
Informal housing	89 (40·8%)	150 (40·2%)	138 (35·1%)	103 (36·8%)	480 (38·0%)
Subdistrict type (N=1246)					
Ger	107 (48·6%)	173 (46·9%)	167 (43·6%)	124 (45·3%)	571 (45·8%)
Apartment	34 (15·5%)	63 (17·1%)	63 (16·4%)	26 (9·5%)	186 (14·9%)
Mixed	79 (35·9%)	133 (36·0%)	153 (39·9%)	124 (45·3%)	489 (39·2%)
Currently breastfeeding (N=1264)	153 (70·5%)	222 (59·5%)	210 (53·4%)	153 (54·4%)	738 (58·4%)
Oral antibiotics within 48 hours prior to admission (N=1277)	121 (55·3%)	187 (48·6%)	178 (45·4%)	131 (46·6%)	617 (48·3%)
Received hospital antibiotics prior to swab (N=1239)	100 (46·5%)	183 (49·3%)	176 (47·2%)	133 (47·5%)	592 (47·8%)
PCV13 vaccination status‡ (N=1186)					
Under-vaccinated	220 (100·0%)	201 (59·1%)	156 (43·3%)	100 (37·6%)	677 (57·1%)
Vaccinated	0 (0·0%)	139 (40·9%)	204 (56·7%)	166 (62·4%)	509 (42·9%)
Pneumococcal carriage (N=1283)	109 (49·5%)	200 (51·7%)	184 (46·7%)	128 (45·4%)	621 (48·4%)
*lyt*A positive samples, serotype unknown§ (N=621)	12 (11·0%)	31 (15·5%)	26 (14·1%)	20 (15·6%)	89 (14·3%)
Vaccine-type carriage (N=1194)	72 (34·6%)	94 (26·4%)	66 (17·9%)	38 (14·5%)	270 (22·6%)

* Cold season refers to the winter months (Nov-March) and warm season refers to non-winter months.
† See supplementary figure 3 for histograms of age distribution by year of enrolment
The definition of minimum living standard was changed by the Government of Mongolia between in 2017 from 170 000₮ to 241 000₮ per person/per month
‡ Children are considered vaccinated if they have received at least two doses when administered at less than 12 months of age or at least one dose when administered at greater than or equal to 12 months of age.
§ Serotype unknown because the sample was culture negative or low DNA yield

Supplementary figure 3: Age distribution of participants that were tested for pneumococcal carriage by year of enrolment, Ulaanbaatar, Mongolia, November 2015-March 2019

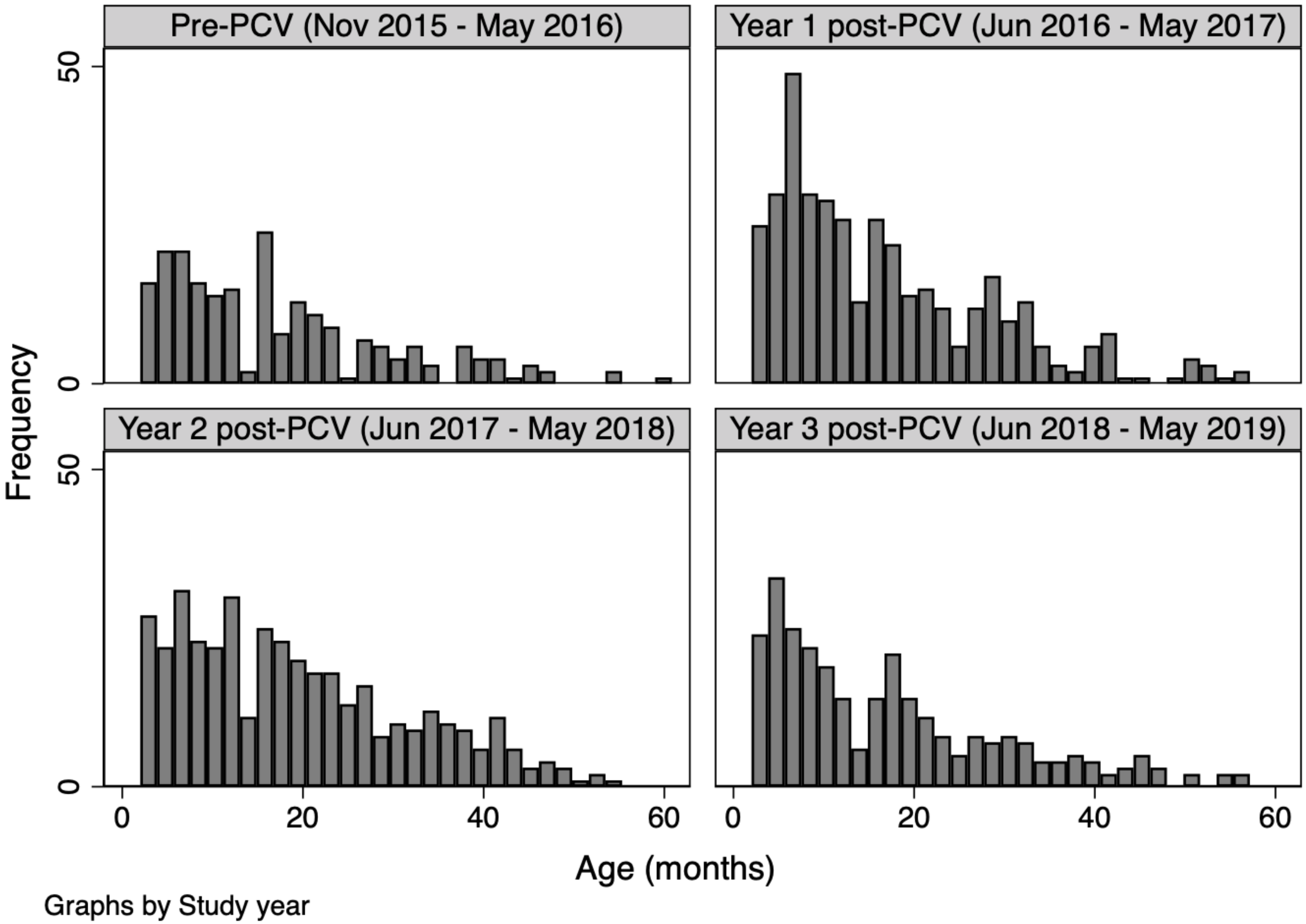

Pre-PCV (Nov 2015 - May 2016)
Year 1 post-PCV (Jun 2016 - May 2017)
Year 2 post-PCV (Jun 2017 - May 2018)
Year 3 post-PCV (Jun 2018 - May 2019)
Frequency
0
50
0
50
0
20
40
60
0
20
40
60
Age (months)
Graphs by Study year

Supplementary Table 5: Pneumococcal carriage by age group, vaccination status and year of enrolment, Ulaanbaatar, Mongolia, November 2015-March 2019

	Pre-PCV (Nov 2015 - May 2016)	Year 1 post-PCV (Jun 2016 - May 2017)	Year 2 post-PCV (Jun 2017 - May 2018)	Year 3 post-PCV (Jun 2018 – May 2019)	Total (Nov 2015 - Mar 2019)
	N=220	N=387	N=394	N=282	N=1283
Pneumococcal carriage					
2-11 months (N=519)	48 (53.3%)	89 (52.4%)	64 (48.1%)	57 (45.2%)	258 (49.7%)
12-23 months (N=411)	40 (52.6%)	62 (52.1%)	59 (44.0%)	39 (47.6%)	200 (48.7%)
24-59 months (N=353)	21 (38.9%)	49 (50.0%)	61 (48.0%)	32 (43.2%)	163 (46.2%)
Vaccine-type carriage					
2-11 months (N=482)	36 (40.9%)	36 (23.2%)	21 (16.8%)	22 (19.3%)	115 (23.9%)
12-23 months (N=381)	24 (35.3%)	30 (27.3%)	24 (19.0%)	9 (11.7%)	87 (22.8%)
24-59 months (N=331)	12 (23.1%)	28 (30.8%)	21 (17.9%)	7 (9.9%)	68 (20.5%)
Vaccine-type carriage, vaccinated					
2-11 months (N=175)	-	10 (18.2%)	7 (11.5%)	12 (20.3%)	29 (16.6%)
12-23 months (N=205)	-	12 (19.0%)	13 (15.7%)	6 (10.2%)	31 (15.1%)
24-59 months (N=95)	-	1 (10.0%)	8 (16.7%)	4 (10.8%)	13 (13.7%)
Vaccine-type carriage, undervaccinated					
2-11 months (N=279)	36 (40.9%)	22 (25.3%)	13 (24.1%)	10 (20.0%)	81 (29.0%)
12-23 months (N=146)	24 (35.3%)	11 (34.4%)	8 (25.8%)	3 (20.0%)	46 (31.5%)

24-59 months (N=207)	12 (23.1%)	22 (32.8%)	12 (19.7%)	2 (7.4%)	48 (23.2%)
Non-vaccine-type carriage					
2-11 months (N=482)	17 (19.3%)	43 (27.7%)	39 (31.2%)	26 (22.8%)	125 (25.9%)
12-23 months (N=381)	12 (17.6%)	27 (24.5%)	32 (25.4%)	26 (33.8%)	97 (25.5%)
24-59 months (N=331)	10 (19.2%)	17 (18.7%)	33 (28.2%)	22 (31.0%)	82 (24.8%)

Pneumococcal serotypes by year

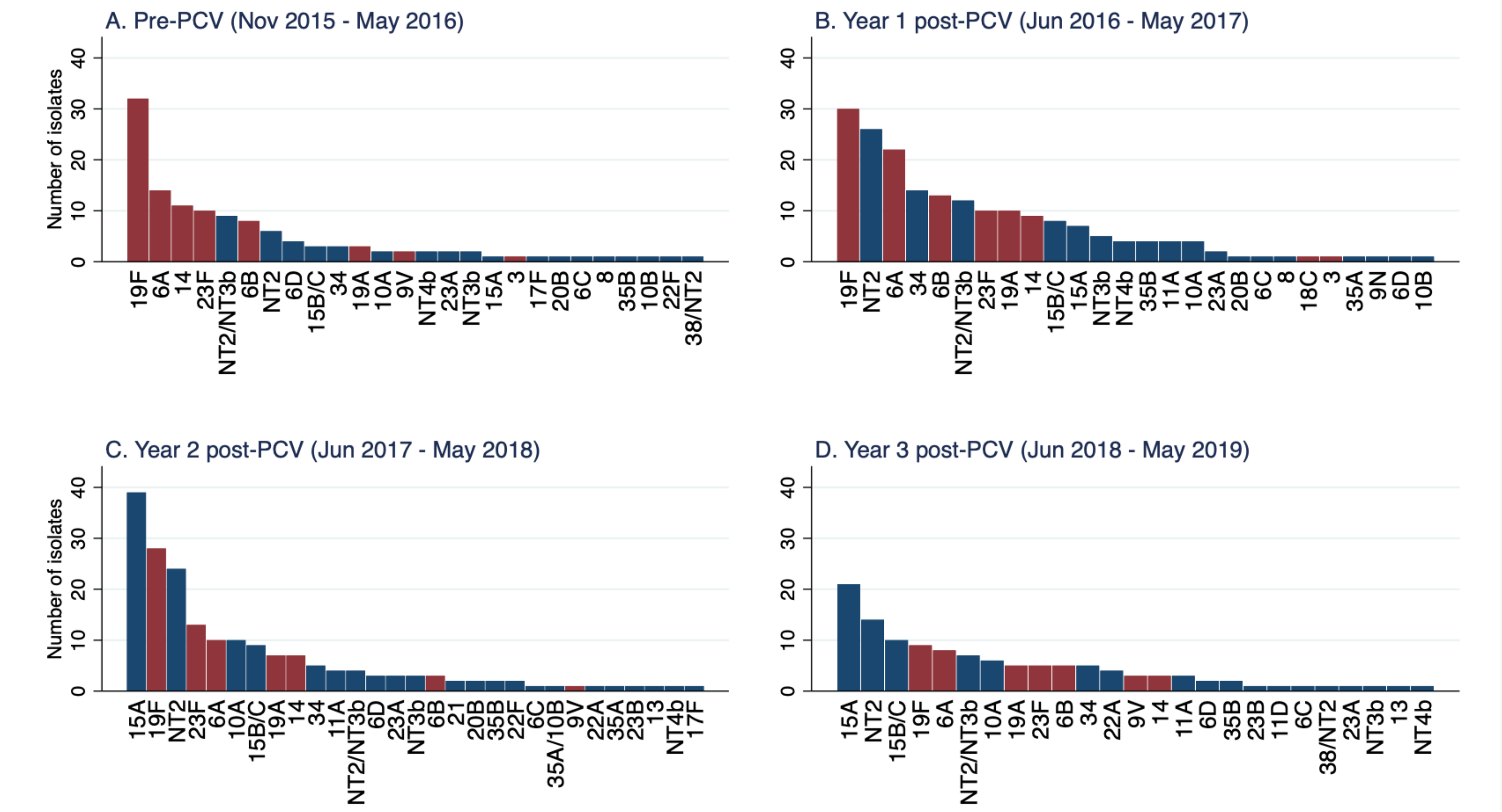

Supplementary Figure 2: All pneumococcal serotypes* detected by year and vaccine-type (red bars – vaccine-types, blue bars – non-vaccine-types), Ulaanbaatar, Mongolia, 2015 – 2019

* Some serotypes are reported together (e.g. 15B/C) as these serotypes are known to interconvert; non-encapsulated pneumococci are reported as NTs and are further classified into different genetic lineages

Antimicrobial resistance genes

Since hospital-based carriage surveillance among children with pneumonia may be affected by prior antibiotic use, we presented supplementary data on the presence of AMR genes. We reported AMR prevalence by vaccination status and over time to assess internal validity and compare data against surveys in non-hospitalised children in the discussion to assess external validity.

Microarray detects ten antimicrobial resistance (AMR) genes associated with mobile genetic elements, encoding tetracycline (*tetM*, *tetK*, *tetO*, *tetL*), chloramphenicol (*cat*), macrolides (*mefA*, *ermB*, *ermC*), kanamycin (*aphA3*), streptothricin (*sat4*), lincosamides (*ermB*, *ermC*), and streptogramin B (*ermB*, *ermC*) resistance. As per previous analyses, we restricted analysis to samples containing a single pneumococcal type with no other species.[21]

AMR genes were common, with 89·1% of pneumococcal positive single-serotype samples containing at least one of the 10 AMR genes assessed.

Supplementary Table 6: Percentage of pneumococcal single-serotype samples with at least one antimicrobial resistance (AMR) gene by vaccination status and study year, Ulaanbaatar, Mongolia, 2015-2019

	Percentage of pneumococcal single-serotype samples with at least one AMR gene (N=450)
Total	401 (89·1%)
PCV13 vaccination status	
Under-vaccinated	222 (90·2%)
Vaccinated	147 (87·5%)
Missing vaccination status	32 (88·9%)
Study year	
Pre-PCV (Nov 2015 - May 2016)	67 (89·3%)
Year 1 post-PCV (Jun 2016 - May 2017)	131 (89·7%)
Year 2 post-PCV (Jun 2017 - May 2018)	118 (90·1%)
Year 3 post-PCV (Jun 2018 – May 2019)	85 (86·7%)

Association between PCV13 coverage and indirect effects

Supplementary Table 7 provides the raw numbers of participants available for Aim 2 analyses – examining the association between PCV13 coverage and indirect effects against VT carriage. Participants stratified by quartiles of PCV13 coverage. There is a trend towards decreasing carriage prevalence among both vaccinated and under-vaccinated participants from the second (25-49%) and third (50-75%) quartiles of PCV coverage compared to the first quartile (<25%). There were few enrolled participants from districts and time periods where PCV13 coverage was greater than 75%

Supplementary Table 7: Vaccine-type pneumococcal carriage prevalence among participants from quartiles of district 13-valent pneumococcal conjugate vaccine (PCV13) coverage, Ulaanbaatar, Mongolia, 2015-2019

	Vaccine-type pneumococcal carriage		
Levels of PCV13 coverage* at each participants' district of residence at the time of enrolment	All participants n/N (%)	Vaccinated participants n/N (%)	Under-vaccinated participants n/N (%)
<25%	91/260 (35·0)	2/3 (66·7)	79/231 (34·2)
25-49%	106/455 (23·3)	27/160 (16·9)	62/220 (28·2)
50-74%	68/460 (14·8)	26/212 (12·3)	28/143 (19·6)
>75%	5/19 (26·3)	2/10 (20·0)	1/3 (33·3)

* Coverage is defined as proportion of children under five years of age who have received at least two doses of PCV13
